# A C-terminally truncated form of β-catenin acts as a novel regulator of Wnt/β-catenin signaling in planarians

**DOI:** 10.1371/journal.pgen.1007030

**Published:** 2017-10-04

**Authors:** Hanxia Su, Miquel Sureda-Gomez, Neus Rabaneda-Lombarte, Maria Gelabert, Jianlei Xie, Wei Wu, Teresa Adell

**Affiliations:** 1 MOE Key Laboratory of Protein Science, School of Life Sciences, Tsinghua University, Beijing, China; 2 Departament de Genètica, Microbiologia i Estadística,Facultat de Biologia, Universitat de Barcelona and Institut de Biomedicina de la Universitat de Barcelona (IBUB), Universitat de Barcelona, Barcelona, Catalunya, Spain; University of Michigan, UNITED STATES

## Abstract

β-Catenin, the core element of the Wnt/β-catenin pathway, is a multifunctional and evolutionarily conserved protein which performs essential roles in a variety of developmental and homeostatic processes. Despite its crucial roles, the mechanisms that control its context-specific functions in time and space remain largely unknown. The Wnt/β-catenin pathway has been extensively studied in planarians, flatworms with the ability to regenerate and remodel the whole body, providing a ‘whole animal’ developmental framework to approach this question. Here we identify a C-terminally truncated *β-catenin* (*β-catenin4*), generated by gene duplication, that is required for planarian photoreceptor cell specification. Our results indicate that the role of β-catenin4 is to modulate the activity of β-catenin1, the planarian β-catenin involved in Wnt signal transduction in the nucleus, mediated by the transcription factor TCF-2. This inhibitory form of β-catenin, expressed in specific cell types, would provide a novel mechanism to modulate nuclear β-catenin signaling levels. Genomic searches and *in vitro* analysis suggest that the existence of a C-terminally truncated form of β-catenin could be an evolutionarily conserved mechanism to achieve a fine-tuned regulation of Wnt/β-catenin signaling in specific cellular contexts.

## Author summary

The Wnt signaling pathway is essential for proper intercellular communication in every developmental process since it controls basic cellular events as cell fate or proliferation. The key element of the Wnt signaling is β-catenin, which controls the transcription of multiple genes in the Wnt receiving cell. A main level of regulation of the Wnt/β-catenin signaling occurs in the cytoplasm, where β-catenin protein levels depend on the activity of the β-catenin destruction complex. However, once it reaches the nucleus, *β-catenin* transcriptional activity requires a fine-tuned regulation to enable the multiple context-specific responses that it performs. These nuclear mechanisms that regulate the Wnt/β-catenin signaling remain poorly understood. Here we report the existence of C-terminal truncated forms of *β-catenin* in planarians (*β-cat3* and *4*), which, *in vitro*, do not show transactivation activity and compete with the canonical planarian *β-catenin* (*β-cat1*), thus acting as competitor inhibitors. Functional analyses in planarians indicate that β-cat4 acts as a negative regulator of β-cat1 during planarian eye photoreceptor specification. We provide evidence to suggest that this novel mechanism for the regulation of nuclear β-catenin activity could be conserved across animal evolution.

## Introduction

The Wnt/β-catenin pathway is an evolutionarily conserved intercellular signaling pathway with essential roles in virtually every developmental process [1–3] and links to a wide range of human diseases [3–7]. Given its multiple, context-dependent roles, the pathway must be extensively regulated. A key element of this pathway is β-catenin, a bi-functional protein first discovered as a component of adherens junctions [8, 9]. β-catenin transduces the Wnt signal to the nucleus [5, 10] and is primarily regulated at the level of nuclearization. Binding of Wnts to their receptors (Frizzleds and LRP5/6) uncouples the β-catenin destruction complex (mainly composed of APC [adenomatous polyposis coli], Axin, CK-1 and GSK-3) and promotes β-catenin stabilization and its nuclear translocation [5, 11–14]. Inhibition of the ligand-receptor interaction through secreted inhibitory molecules (WIF, sFRP, DKK) also represents a common level of Wnt/β-catenin signal regulation [15–18]. Since β-catenin does not have a DNA binding domain, once it reaches the nucleus it must interact with a member of the DNA‑binding T cell factor/lymphoid enhancer factor (TCF/LEF) family to regulate its downstream targets [19, 20]. Several β-catenin/TCF partners have been identified, which mainly target the C-terminal part of β-catenin, and which confer master regulatory properties to β-catenin since they are mainly involved in regulating chromatin structure and RNA polymerase II [21, 22]. Thus, the final activity of β-catenin relies not only on its nuclearization but also on its ability to bind to TCFs and their nuclear co-factors. Although an increasing number of factors have been reported to modulate the transcriptional activity of the β-catenin/TCF complex (e.g. ICAT, Groucho and Chibby) [20, 22–27], the regulation of β-catenin activity once it reaches the nucleus remains poorly understood.

Planarians, flatworms with an almost unlimited ability to regenerate and remodel their tissues during their whole life span [28–30], have become a robust model to study the function of Wnt/β-catenin signaling in different developmental contexts [31–39]. Although most organisms have a single bi-functional β-catenin protein, gene duplication and functional specialization have led to the generation of two β-catenins in planarians: Smed-β-catenin1 (β-cat1) and Smed-β-catenin2 (β-cat2) [40]. β-cat1 is the intracellular effector of Wnt/β-catenin signaling but exerts no role in cell adhesion, whereas β-cat2 is exclusively found in cell-cell junctions [40]. This functional diversification provides an ideal scenario in which to study the Wnt signaling properties of β-catenin. A functional specialization of β -catenins has also been found in the nematode *C*. *elegans*. However, their specific role in nuclear signaling appears extremely complex and, apparently, divergent [41, 42]. Thus, although genetic tools to generate cells that exclusively lack canonical Wnt pathway activity have been reported in mouse [43], planarians represent an excellent scenario in which to study the signaling properties of β-catenin in vivo without interference of the cell adhesion properties.

Functional analysis of *β-cat1* and the main elements of the Wnt/β-catenin signaling pathway demonstrate an essential role for this pathway in the specification of the antero-posterior (A-P) axis during planarian regeneration and homeostatic cell turnover [31–34, 44]. *β-cat1* silencing generates a range of anteriorized phenotypes, from “tailless” to “radial-like hypercephalized” planarians [32, 45]. Recently, novel functions for *β-cat1* have been reported in planarian brain and eye regeneration, and in gonad development [34, 36, 46]. Importantly, analysis of β-cat1 protein localization reveals that it is present in the nucleus of posterior cells, according to its role in A-P axial identity specification, and also in the main planarian tissues [45]. Thus, given that it has both activity- and context-dependent effects, nuclearization alone cannot account for its regulation. This makes planarians an excellent model to further understand how the transcriptional activity of β-catenin might be regulated once it is in the nucleus.

Here we report the existence of two new planarian β-catenins, *β-catenin3* (*β-cat3*) and *β-catenin4* (*β-cat4*), which have a truncated C-terminal transactivation domain and are expressed primarily in the nervous system. β-cat3 and β-cat4 can bind to TCF but do not activate the Wnt signal in vitro. Functional analysis in planarians indicates that β-cat4 acts as a negative regulator of nuclear β-cat1 in planarian eye photoreceptors probably by competing for binding to TCF-2, a new TCF found in planarian photoreceptor cells. We provide evidence to suggest that this novel mechanism for the regulation of nuclear β-catenin activity could be conserved across animal evolution.

## Results

### Smed-β-catenin3 and 4 are two new β-catenin homologs

**Fig 1:**
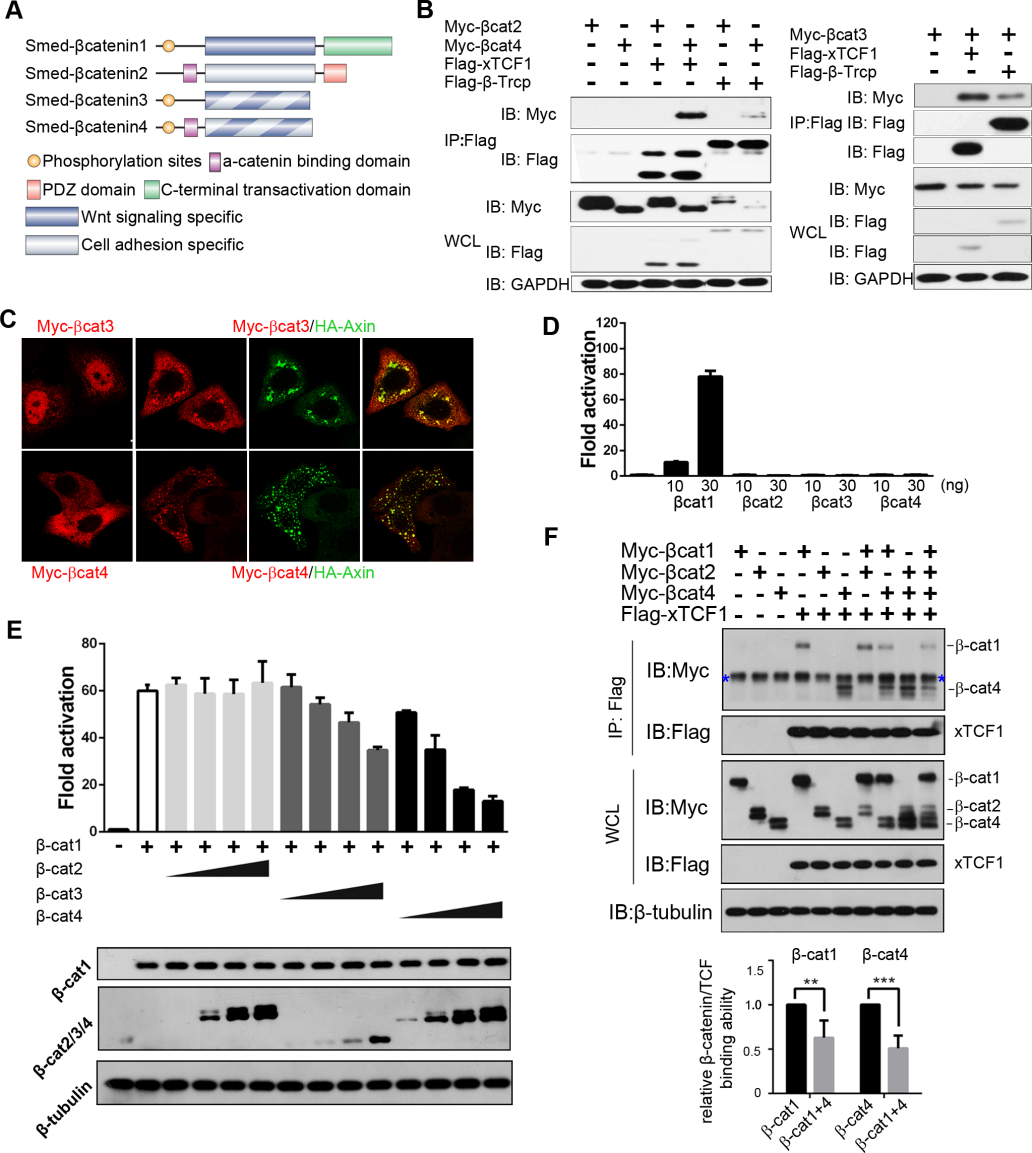
β-cat3 and 4 inhibit β-cat1 dependent Wnt signaling. **(A)** Schematic of the four *β-catenin* homologs in *S*. *mediterranea*. β-cat1 and β-cat2 are structurally segregated. β-cat1 conserves the N-terminal GSK3 phosphorylation sites, the binding surface for Wnt signaling components in the central armadillo repeats, and the C-terminal transactivation domain. β-cat2 conserves the N-terminal α-catenin binding motif, the interacting platform for the cadherin complex in the central armadillo repeats but not for Wnt signaling elements, and the C-terminal PDZ domain. The newly identified β-cat3 and 4 conserve the GSK3 phosphorylation sites, the binding surface for Wnt signaling components and a partial conservation of the cell adhesion elements, whereas they have lost the C-terminal transactivation domain. (**B)** HEK293T cells were transfected with the indicated plasmids, and lysates were immunoprecipitated (IP) with FLAG-M2 beads. Western blotting (IB) with anti-Myc revealed co-IP of β-cat3 and 4 following FLAG-M2 immunoprecipitation, indicating that both of them interact with TCF and β-Trcp. β-cat2 was analyzed as a negative control. **(C)** Localization of β-cat3 and 4 alone or co-expressed with Axin in transfected HeLa cells. In HeLa cells, β-cat3, which localized mainly in the nucleus alone, was recruited to the cytoplasm when co-transfected with Axin. β-cat4, which was more widely dispersed in the cytoplasm, was recruited by Axin and had a punctate distribution. Scale bar = 20 μm. **(D)** TOPflash reporter assay following co-transfection of HEK293T cells with β-cat1, β-cat2, β-cat3, β-cat4 and reporter plasmids.10 or 30 ng were transfected of each β-catenin. Just β-cat1 but not β-cat3 or 4 activated Wnt/β-catenin signaling. β-cat2 does not show Wnt reporter activity, as reported [40]. **(E)** TOPflash reporter assay following co-transfection of HEK293T cells with β-cat1 (20 ng), β-cat2 (10, 20, 30 or 60 ng), β-cat3 (10, 20, 30 or 60 ng), β-cat4 (10, 20, 30 or 60 ng) and reporter plasmids. The co-transfection of β-cat3 or 4 inhibited β-cat1 reporter activation in a dosage dependent manner. Immunoblot analysis shows the protein expression level of β-cat1/2/3/4 in each line. **(F)** HEK293T cells were transfected with the indicated plasmids, and lysates were immunoprecipitated (IP) with FLAG-M2 beads. Western blot was performed with anti-Myc and anti-Flag antibodies. Upon co-transfection of β-cat1 and β-cat4, the amount of immunoprecipitated β-catenins by TCF was less than that during sole transfection of either, supporting that they compete with each other for TCF binding. The relative protein levels of precipitated β-catenins by TCF were quantified and normalized against total β-catenins in WCL (whole cell lysates). Quantitative results of relative binding of β-cat1 or β-cat4 alone with respect to β-cat1+β-cat4 derived from four independent experiments. β-cat1, 0.63±0.19; β-cat4, 0.51±0.14 (SD; n = 4). **p<0.05, ***p<0.001 (t test). Relative binding of β-cat1 or β-cat4 alone with respect to β-cat1+β-cat2 or β-cat4+β-cat2, respectively, was measured as a negative control. β-cat1, 1.11; β-cat4, 0.91 (n = 1). Blue asterisk indicates non-specific bands.

A search for *β-catenin* family members in the *Schmidtea mediterranea* transcriptomes revealed two new genes with protein sequences indicating that they were *β-catenin* homologs (S1 Fig). We named them *Smed-β-catenin3* (*β-cat3*) and *Smed-β-catenin4* (*β-cat4*), since two *β-catenin* paralogs had been already reported in this species [31–33, 40]. β-cat3 and 4 proteins conserve the GSK3 phosphorylation sites in the N-terminal region, and their armadillo repeats contain the interacting amino acids for multiple β-catenin-binding proteins, including APC, Axin, TCF and E-cadherin (Fig 1A and S1 Fig). The α-catenin binding sites are conserved in β-cat4 but not in β-cat3 (Fig 1A and S1 Fig). Importantly, the C-terminal transactivation domain, which interacts with crucial chromatin‑dependent factors, is lost in both β-catenins (Fig 1A and S1 Fig). The finding that the Wnt signaling domains but not the transactivation domain are conserved suggests that β-cat3 and 4 could function as dominant-negative forms of β-cat1, which is the β-catenin homolog involved in signaling to the nucleus in planarians [37, 40].

### β-cat3 and 4 inhibit β-cat-dependent Wnt signaling *in vitro*

Since β-cat3 and 4 contain several conserved domains or residues involved in Wnt signaling and cell adhesion, we further tested the interaction of β-cat3 and 4 with the main components involved in these two processes in mammalian cell lines. Co-immunoprecipitation experiments indicated that β-cat4 strongly interacts with the cell-cell adhesion elements E-cadherin and α-catenin (S2A Fig), whereas β-cat3 showed interaction with E-cadherin but not α-catenin (S2B Fig). This observation is consistent with the conservation of their functional protein domains (Fig 1A and S1 Fig). In this experiment, β-cat1 was used as a control, since it retains the essential residues for the interaction with E-cadherin, but not the conserved α-catenin binding domain [40]. Interestingly, both β-cat3 and 4 were able to interact with the elements involved in Wnt signaling, β-Trcp and TCF (Fig 1B). In this experiment, planarian β-cat2 was used as a control and, according to its reported role in cell adhesion but not in the Wnt cascade [40], it showed no binding to β-Trcp or TCF. Moreover, immunofluorescence assays revealed co-localization of β-cat3 and 4 with Axin, the core element of the β-catenin destruction complex, which is also consistent with their protein sequence analysis (Fig 1C).Thus, these results indicate that β-cat3 and 4 are under the control of the β-catenin destruction complex, and have the potential to bind to their nuclear co-factor TCF.

Considering the loss of their C-terminal transactivation domain, our results further support the hypothesis that β-cat3 and 4 could act as dominant negative forms of β-cat1. To test this hypothesis, we used the Super-TOPflash reporter system in HEK293T cells [47] to analyze the potential of β-cat3 and 4 to activate Wnt/β-catenin signaling. Whereas β-cat1 activated the reporter significantly, consistent with its reported role in Wnt signal transduction [40], β-cat3 and 4 had no effect on the reporter, even after increasing the dosages (Fig 1D). Consistent with its specific role in cell adhesion [40], the β-cat2 paralog was also not able to activate the Super-TOPflash reporter. Importantly, when β-cat1 was co-transfected together with β-cat3 or 4, the levels of reporter activity decreased in a dose-dependent manner (Fig 1E). The same result was obtained when analyzing the axial induction capability of planarian *β-catenin*s in *Xenopus* embryos (S2C Fig).

To further test whether β-cat3/4 could act as competitors of β-cat1 for the binding to TCF, we performed a binding competition assay. Following co-transfection of HEK293T cells with β-cat1, β-cat4 and TCF, quantitative analysis indicated that β-cat1 and β-cat4 disrupt each other’s binding to TCF (Fig 1F). The specificity of this competition is supported by the finding that co-transfection of β-cat2 does not alter the binding of β-cat1 or 4 to TCF (Fig 1F).

These results demonstrate that β-cat3 and 4 do not show any transactivation properties and that their expression inhibits β-cat1 activity ‘in vitro’ or in a heterologous system. Furthermore, both β-cat4 and β-cat1 are able to bind to TCF. These results are consistent with a role of β-cat3 and 4 as competitive inhibitors of β-cat1.

### *β-cat4* is required for photoreceptor specification during planarian regeneration and homeostasis

**Fig 2:**
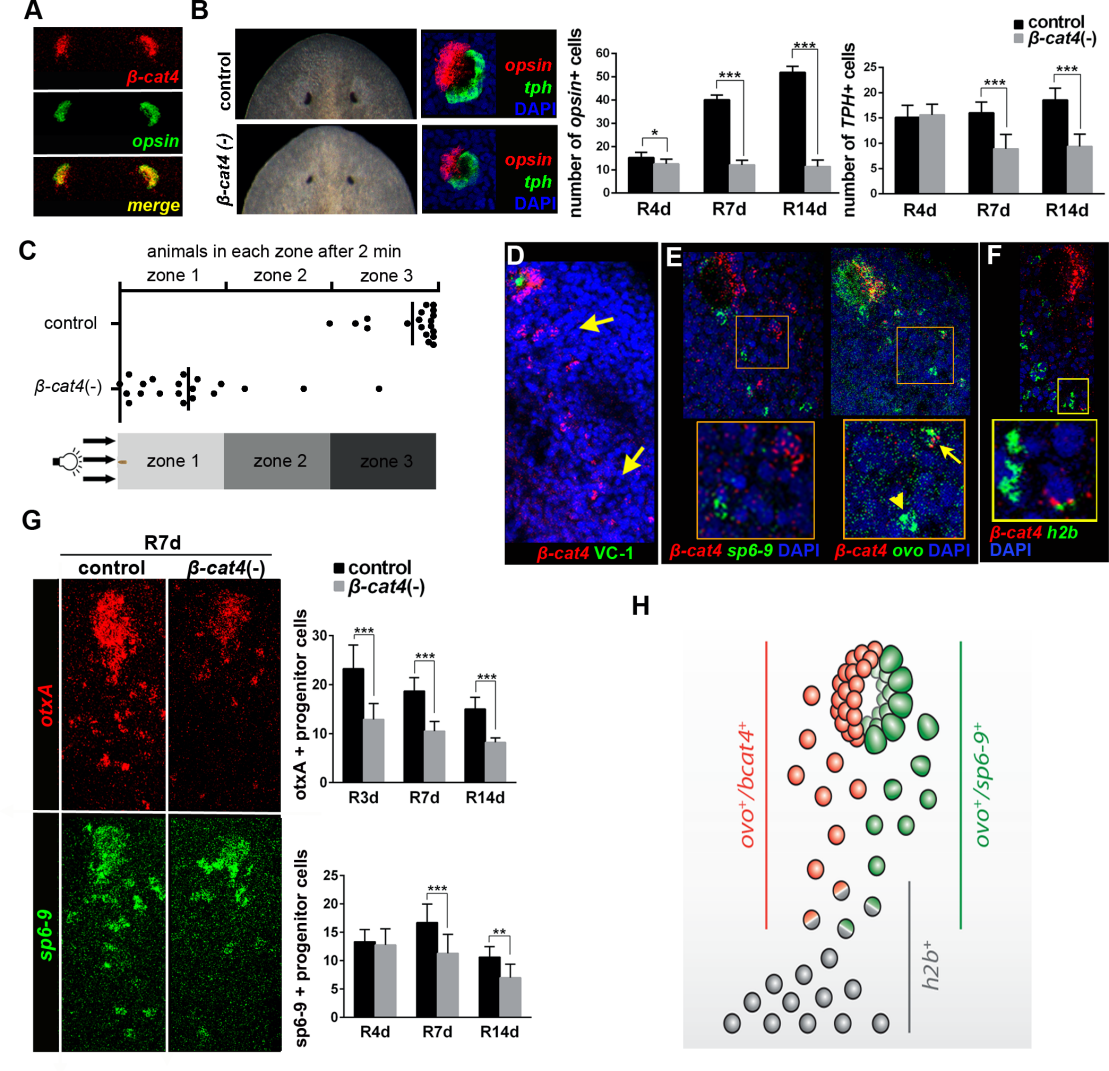
*Smed-β-cat4* is required for planarian photoreceptor specification. **(A)** Double FISH assay with *β-cat4* (red) and *opsin* (green), a marker of photoreceptor cells, in intact animals, showing their co-localization. **(B)** Left, live and FISH [*opsin* (red) and *tph* (green)] images of control and *β-cat4* (RNAi) planarian eyes at 12 days of regeneration. Right, quantification of the number of photoreceptor (*opsin+*) and pigment (*tph+*) cells per eye at 4, 7 and 14 days of regeneration in control and *β-cat4* (RNAi) planarians. *opsin*+ cells in control R4d, 15.25±2.19 (SD; n = 8 eyes); *β-cat4* (RNAi) R4d,12.5±2.07 (SD; n = 8 eyes); control R7d,40.11±2.03 (SD; n = 9 eyes); *β-cat4* (RNAi) R7d,12.08±1.98 (SD; n = 12 eyes); control R14d,51.92±2.50 (SD; n = 12 eyes); *β-cat4* (RNAi) R14d,11.38±2.83 (SD; n = 8 eyes). *tph*+ cells in control R4d, 15.13±2.36 (SD, n = 8 eyes); *β-cat4* (RNAi) R4d, 15.63±2.07 (SD; n = 8 eyes); control R7d, 16.00±2.12 (SD; n = 9 eyes); *β-cat4* (RNAi) R7d, 8.92±2.81 (SD; n = 12 eyes); control R14d, 18.58±2.27 (SD; n = 12 eyes); *β-cat4* (RNAi) R14d, 9.38±2.45 (SD; n = 8 eyes). *p<0.05, ***p<0.001 (t test). **(C**) Phototaxis assay of *β-cat4* (RNAi) animals. Graphical representation of the percentage of control and *β-cat4* (RNAi) planarians found in the different regions in 2 minutes (n = 20). The schematic of the container indicates the clearest and the darkest zone. **(D)** *β-cat4* (red) FISH followed by immunohistochemistry with VC-1 (anti-arrestin) antibody (green), to label the visual axons, shows the expression of *β-cat4* in a trail of isolated cells posterior to the eyes (yellow arrows) in addition to expression in the photoreceptors in the eyes. Animals were analyzed at 4 days of regeneration. **(E)** Double FISH of *β-cat4* (red) and *ovo* (green), a pan-eye cell marker, or *sp6-9* (green), a pigment progenitor cell marker, shows that among all ovo+ cells some of them co-express with *β-cat4* (ovo+*/β-cat4+*, yellow arrow) and some of them do not (ovo+*/β-cat4-*, yellow arrowhead). *β-cat4* was never co-expressed with *sp6-9*. Insets show magnifications of the selected areas. Animals were analyzed at 7 days of regeneration. **(F)** Double FISH of *β-cat4* (red) and *h2b* (green) shows that *β-cat4* is expressed in stem cells. Animals were analyzed at 7 days of regeneration. **(G)** FISH of *otxA* (red), a photoreceptor progenitor cells marker, and *sp6-9* (green), in control and *β-cat4* (RNAi) animals, at 7 days of regeneration. The quantification of *otxA*+ and *sp6-9*+ cells in the trail posterior to the eyes at different regeneration time points is shown. *otxA*+ cells in control R3d, 23.25±4.81 (SD; n = 13 eyes; *β-cat4* (RNAi) R3d, 12.89±3.26 (SD; n = 9 eyes); control R7d, 18.69±2.72 (SD; n = 13 eyes); *β-cat4* (RNAi) R7d, 10.50±1.96 (SD; n = 10 eyes); control R14d, 15.00±2.39 (SD; n = 8 eyes); *β-cat4* (RNAi) R14d, 8.20±0.92 (SD; n = 10 eyes). *sp6-9*+ cells in control R4d, 13.33±2.16 (SD; n = 6 eyes); *β-cat4* (RNAi) R4d, 12.75±2.87 (SD; n = 8 eyes); control R7d, 16.71±3.25 (SD; n = 7 eyes); *β-cat4* (RNAi) R7d, 11.29±3.35 (SD; n = 7 eyes); control R14d, 10.63±1.85 (SD; n = 8 eyes); *β-cat4* (RNAi) R14d, 7.00±2.40 (SD; n = 9 eyes). **p<0.01, ***p<0.001 (t test). **(H)** Schematic of *β-cat4* expression in the eyes. Stem-cells (*h2b+*) acquire expression of *ovo* to become eye progenitors. Eye progenitors became photoreceptor or pigment cells when acquiring the expression of *β-cat4* or sp6-9, respectively. In all images anterior is to the top. Scale bars, 50 μm (A-F), and 10 μm (E and F insets).

Since β-cat3 and 4 act through inhibition of β-cat1, they could be essential in any of the processes in which β-cat1 is involved, such as posterior identity specification or organogenesis [31–34, 36, 45, 46, 48]. Whole-mount *in situ* hybridization (WISH) in intact and regenerating animals showed that *β-cat3* and *4* are expressed in the parenchyma and in the central nervous system of intact animals, as well as in the new regenerating brain (S3A and S3B Fig). Remarkably, *β-cat4* is also highly expressed in the eyes (S3A and S3B Fig), specifically in photoreceptors, since fluorescent *in situ* hybridization (FISH) analysis demonstrated that it is exclusively expressed in *opsin*+ cells (Fig 2A) [49]. No apparent defects were observed in regenerating *β-cat3* (RNAi) planarians (S3C Fig). In contrast, compared to control animals, *β-cat4* (RNAi) animals regenerated smaller eyes, with smaller pigmented spots and missing the periglobular unpigmented epidermis, which corresponds to the photoreceptor area (Fig 2B). Importantly, posterior identity specification, which is disrupted in *β-cat1* (RNAi) animals [31–33], was not affected after *β-cat4* (RNAi) (S3C and S3D Fig). The efficiency and specificity of the RNAi inhibition was assessed by qPCR, showing that *β-cat4* RNAi animals show highly reduced levels of *β-cat4* but not of *β-cat1*, *2* and *3* mRNA (S3E Fig). Thus, we focused on the study of *β-cat4* function specifically in the eye.

Planarian eyes are simple structures comprising two main, well-characterized cell types: photoreceptors and pigment cells [50, 51]. The number of each cell type was quantified during regeneration of *β-cat4* (RNAi) planarians by analyzing the expression of *opsin* and *tph*, specific markers of photoreceptor and pigment cells, respectively [52] (Fig 2B and S4 Fig). Remarkably, RNAi of *β-cat4* resulted in reduced photoreceptor cells early in regeneration (R4d), whereas pigment cells did not show significant differences at this stage (Fig 2B and S4 Fig). As regeneration progressed, *β-cat4* (RNAi) animals showed a significantly reduced number of pigment cells compared to control (Fig 2B and S4 Fig), possibly due to a non-autonomous effect [53]. Consistent with the effects on photoreceptor cells, *β-cat4* (RNAi) animals did not show the proper negative phototaxis behavior (Fig 2C). When exposed to a light gradient, all control animals moved away from the light and remained in the darkest zone (zone 3). Conversely, although *β-cat4* (RNAi) organisms seemed to move normally, most of them remained in the clearest zone and did not reach the darkest zone in the same time period (Fig 2C and S1 and S2 Movies). These data show that *β-cat4* silencing causes a reduction of photoreceptor cells, followed by a reduction of pigment cells, and influences their normal behavioral responses to light, suggesting its role in photoreceptor specification.

Since planarians continuously remodel their tissues [28], we analyzed whether *β-cat4* is also required for eye cell maintenance during normal planarian homeostasis. Injection of *β-cat4* dsRNA over a period of 5 weeks produced a decrease in eye size and in the photoreceptor area (S5A Fig). Quantification of photoreceptor and pigment cells through *opsin* and *tph* FISH over the 5 weeks of the experiment revealed that *β-cat4* (RNAi) animals always have fewer photoreceptor cells (S5A Fig). The gradual reduction of photoreceptor cell number observed in control animals (S5A Fig) is due to shrink age of the animals, which remained starved over the 5-week period. According to the phenotype, *β-cat4* (RNAi) animals showed a defective negative phototaxis response that got worse as the experiment progressed (S5B Fig). Thus, these data demonstrate that *β-cat4* is required for photoreceptor maintenance during homeostasis.

Planarian photoreceptor and pigment cells differentiate from progenitor cells that are located as a trail of cells extending caudally from the eye and express the pan-eye marker *ovo* [54]. A small number of eye progenitor cells co-express *ovo* with stem-cell specific markers (*h2b*) and correspond to the specialized eye stem-cells [54]. Eye stem cells acquire the expression of specific determinants that direct their final fate to photoreceptor or pigment cells [52, 55]. In order to understand the mechanism by which *β-cat4* influences eye regeneration, *β-cat4* expression was further studied with FISH. Besides its expression in eye photoreceptor cells, *β-cat4* was found to be expressed in the trail of eye precursors posterior to the eye both in intact animals and during regeneration (Fig 2D and S5C Fig). Double FISH analysis of *β-cat4* with *ovo* and *sp6-9*, a pigment cell determinant, revealed that *β-cat4* is exclusively expressed in photoreceptor but not pigment-cell progenitors, since it was always co-expressed with the eye marker *ovo* but never with the pigment-specific marker *sp6-9* (Fig 2E and S5D Fig). In addition, a few isolated *β-cat4*+ cells were also found to be *h2b+* (Fig 2F), indicating that *β-cat4* is already expressed in the eye stem cell.

To test whether *β-cat4* is required for specification of photoreceptor cells from the common eye stem cell precursor, we quantified the number of photoreceptor and pigment-cell progenitors in the eye trail of *β-cat4* (RNAi) animals using the specific markers *otxA* and *sp6-9*, which label differentiating photoreceptor and pigment cells, respectively [52, 54, 55]. As expected, *β-cat4* (RNAi) animals had a reduced number of *otxA*+ cells in the eye from early regeneration stage and a later decrease in *sp6-9*+ cells (S6 Fig). Importantly, the same result was observed when quantifying *otxA*+ and *sp6-9*+ cells in the trail; *β-cat4* (RNAi) resulted in failure of photoreceptor progenitor-cell specification from the early regeneration stage (R3d), whereas pigmented cells appeared reduced at a later stage (R7d) (Fig 2G and S6 Fig). Thus, *β-cat4* is required for photoreceptor progenitor-cell specification from the common eye stem cell (Fig 2H).

### *β-cat4* specifies photoreceptor cells through *β-cat1* inhibition

**Fig 3:**
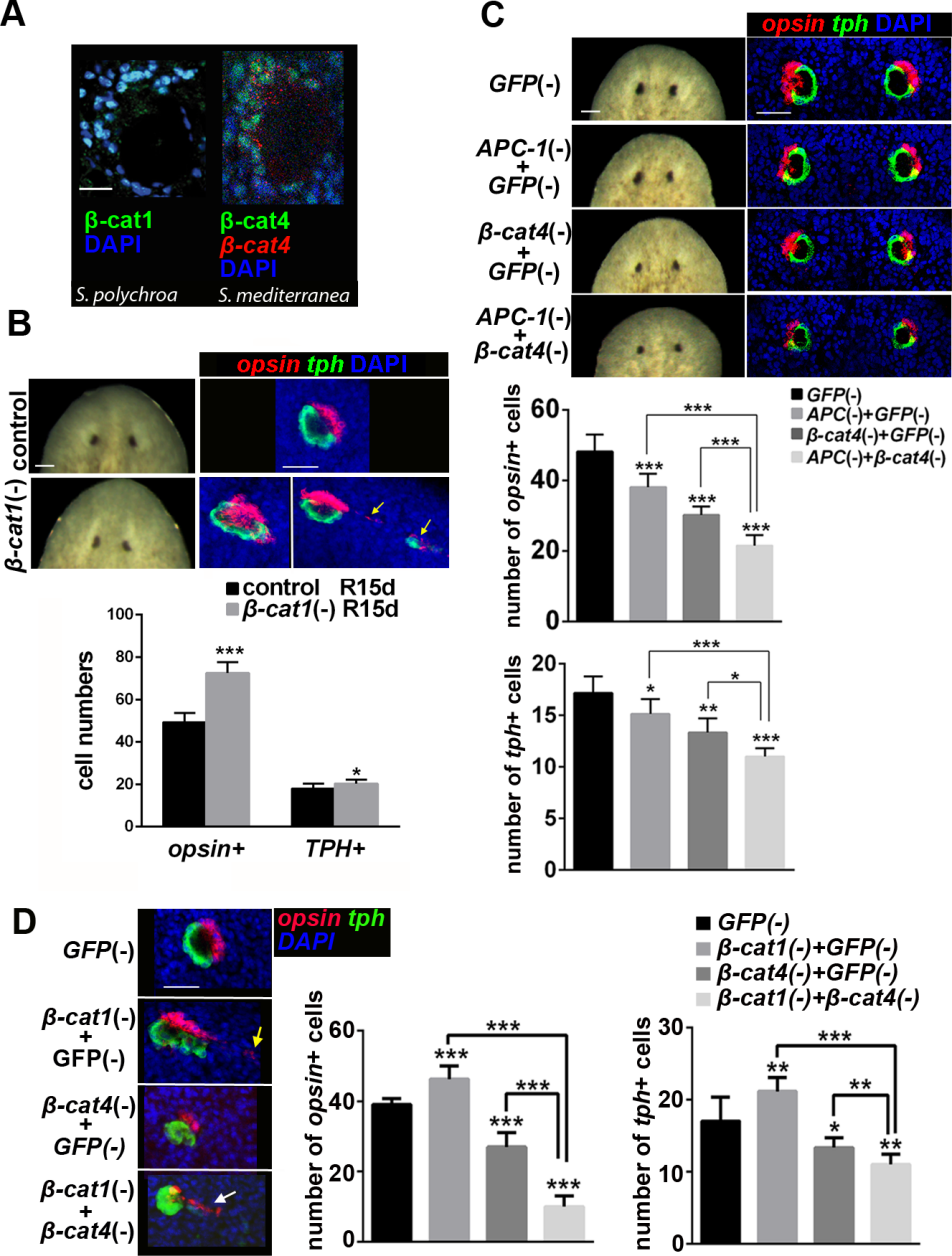
*β-cat4* specifies photoreceptor cells through *β-cat1* inhibition. **(A)** β-cat1 protein (green) localizes to the nucleus of photoreceptors (image corresponds to intact *S*. *polychroa*, syster species of *S*. *mediterranea*). β-cat4 protein (green) and *β-cat4* mRNA (red) colocalize in photoreceptor cells, where the protein is found in the nucleus (images correspond to intact *S*. *mediterranea*) **(B)** Eye phenotype of *β-cat1* (RNAi) animals at 15 days of regeneration. Live images and FISH of *opsin* (red) and *tph* (green). *β-cat1* (RNAi) animals showed elongated and disorganized eyes, with the appearance of ectopic photoreceptor cells in 50% of the eyes analyzed (yellow arrows in the right image). The respective quantification of *opsin*+ and *tph*+ cells per eye is shown. Ectopic cells were not included in the analysis. *opsin*+ cells in control R15d, 49.25±4.37 (SD; n = 12 eyes); *β-cat1* (RNAi) R15d, 72.50±5.21 (SD; n = 6 eyes). *tph*+ cells in control R15d, 18.00±2.26 (SD; n = 12 eyes); *β-cat1* (RNAi) R15d, 20.33±1.86 (SD; n = 6 eyes). *p<0.05, ***p<0.001 (t test). **(C)** Double knockdown of *APC-1* (RNAi) and *β-cat4* (RNAi) in intact animals. Live images and FISH of *opsin* (red) and *tph* (green) after the indicated RNAi treatments with the respective quantification of *opsin*+ and *tph*+ cells per eye. *opsin*+ cells in *GFP* (RNAi), 48.17±4.80 (SD; n = 6 eyes); *APC-1*;*GFP* (RNAi), 38.13±3.76 (SD; n = 8 eyes); *β-cat4*;*GFP* (RNAi), 30.17±2.40 (SD; n = 6 eyes); *APC-1*;*β-cat4* (RNAi), 21.50±2.35 (SD; n = 6 eyes). *tph*+ cells in *GFP* (RNAi), 17.17±1.60 (SD; n = 6 eyes); *APC-1*;*GFP* (RNAi), 15.13±1.46 (SD; n = 8 eyes); *β-cat4*;*GFP* (RNAi),13.33±1.37 (SD; n = 6 eyes); *APC-1*;*β-cat4* (RNAi), 10.83±0.75 (SD; n = 6 eyes). *p<0.05, **p<0.01, ***p<0.001 (t test). *APC-1* (RNAi) and *β-cat4* (RNAi) caused smaller eyes than control. Notice that double RNAi of *APC-1* and *β-cat4* resulted in a more severe phenotype than each one alone. **(D)** Double knockdown of *β-cat1* (RNAi) and *β-cat4* (RNAi). FISH of *opsin* (red) and *tph* (green) after the indicated RNAi treatments in 9 days regenerating animals. The respective quantification of *opsin*+ and *tph*+ cells per eye is shown. *opsin*+ cells in *GFP* (RNAi), 39±1.7 (SD; n = 6 eyes); *β-cat1*;*GFP* (RNAi), 46.25±3.77 (SD; n = 8 eyes); *β-cat4*;*GFP* (RNAi), 27.67±4.03 (SD; n = 6 eyes); *β-cat1*;*β-cat4* (RNAi), 10±3.03 (SD; n = 6 eyes). *tph*+ cells in *GFP* (RNAi), 17±3.34 (SD; n = 6 eyes); *β-cat1*;*GFP* (RNAi), 21.12±1.96 (SD; n = 8 eyes); *β-cat4*;*GFP* (RNAi),13.33±1.37 (SD; n = 6 eyes); *β-cat1*;*β-cat4* (RNAi), 11±1.4 (SD; n = 6 eyes). *p<0.05, **p<0.01, ***p<0.001 (t test). *β-cat1* (RNAi) animals show large eyes with ectopic eye cells (yellow arrow); *β-cat4* (RNAi) animals show small eyes; and double *β-cat1*;*β-cat4* (RNAi) animals show smaller and more disorganized eyes than single *β-cat4* or *β-cat1* (RNAi) (white arrow indicates a row of delocalized photoreceptor and eye cells). Anterior is to the top. Scale bar = 20 μm (A), 50 μm (B, C,D).

Considering that β-cat4 inhibits TCF-mediated β-cat1 activity in cell cultures and that it has lost the C-terminal transactivation domain, β-cat4 could inhibit Wnt signaling in photoreceptors by competing with β-cat1 for TCF binding in the nucleus. To test whether this molecular mechanism could be functional in planarians, we first analyzed *β-cat1* and *β-cat4* expression in the planarian eye field. FISH for *β-cat1* followed by immunostaining of VC-1, which labels the rhabdomeres of photoreceptor cells [52, 56, 57], showed that *β-cat1* was expressed in regenerating photoreceptor cells (S7A Fig). Moreover, double FISH for both *β-cat1* and *β-cat4* mRNA revealed that both are found in photoreceptor cells (S7A Fig). Using a specific antibody generated in this study (S7B Fig) we could demonstrate that β-cat4 protein is localized in the nucleus of photoreceptors (Fig 3A). Immunostaining with a β-cat1-specific antibody [45], revealed that β-cat1 is also localized in the nucleus of photoreceptor cells in *S*. *polychroa*, the sister species of *S*. *mediterranea* (Fig 3A). Thus, our results show that both β-cat1 and β-cat4 are localized in the nucleus of photoreceptor cells, which is consistent with their nuclear interaction.

Next, we performed RNAi experiments to analyze the functional relationship between the two *β-catenin*s. Planarians were decapitated after *β-cat1* dsRNA injection and allowed to regenerate. The efficiency of the inhibition was tested by qPCR (S7C Fig). Newly formed heads showed “slanted eyes” with a very thin and elongated periglobular unpigmented epidermis and pigmented cup (Fig 3B). The observed phenotype is very similar to a previously reported *β-cat1* (RNAi) [38]. FISH with eye-specific markers confirmed that *β-cat1* (RNAi) led to a disordered eye structure, in which photoreceptor and pigment cells formed larger eyes and in which ectopic eye cells appeared (Fig 3B, S7D Fig and S3 and S4 Movies). Quantification of *opsin+* and *tph+* cells present in the eye structure showed an increase with respect to control animals (Fig 3B and S7D Fig). This result is the opposite of that found in *β-cat4* (RNAi) planarians, which had a decrease in the number of photoreceptor cells, thus supporting the opposing role of these *β-catenin*s.

Since techniques for overexpression are currently unavailable in planarians, we took an indirect approach to up-regulate β-cat1 through silencing *APC-1*, the *APC* homolog in planarians [31, 34]. *APC-1* (RNAi) leads to β-cat1 up-regulation and nuclear accumulation [45], resulting in the regeneration of a tail at anterior wounds [31, 34, 45]. In order to analyze the eye field in *APC-1-*knockdown planarians, we performed RNAi experiments in intact animals, since it is known that *APC-1* silencing during two weeks does not lead to tail-head transformation [45]. We injected dsRNA for *APC-1*, *β-cat4*, and *APC-1*;*β-cat4* for 2 weeks. The efficiency of the inhibition was analyzed by qPCR (S7E Fig). As expected, *β-cat4* (RNAi) caused smaller eyes with a decrease in both photoreceptor and pigment cell numbers compared to controls (Fig 3C). Importantly, *APC-1* (RNAi) generated the same phenotype (smaller eyes with fewer photoreceptor and pigment cells) (Fig 3C). Co-silencing *APC-1* and *β-*cat4 caused an even more severe decrease in the number of photoreceptor cells (Fig 3C). This last result is consistent with the hypothesis that *β-*cat4 competes with *β-*cat1 in the nucleus inhibiting its transcriptional activity. However, it should also be considered that, since both *β-*catenins have the potential to be regulated by the destruction complex, in *APC* RNAi animals not only *β-*cat1 but also *β-*cat4 could be stabilized.

*β-cat1* gain of function through *APC-1* (RNAi) results in the same phenotype of diminished eye cell numbers as *β-cat4* (RNAi), whereas *β-cat1* loss of function causes the opposite effect. Thus, β-cat1, as a key downstream transcriptional co-activator in Wnt signaling, plays a negative role in planarian photoreceptor development.

To further understand the functional relationship of β-cat1 and β-cat4 in photoreceptor cells, the phenotype of the double RNAi was analyzed. The efficiency of the inhibition was analyzed by qPCR (S7F Fig). The result shows that co-silencing *β-cat1* and *β-cat4* causes extremely disorganized eyes, with abundant delocalized eye cells, which show a reduction in the number of photoreceptor and pigment cells (Fig 3D). This result does not support the hypothesis of β-cat4 directly acting as a dominant-negative form of β-cat1, but suggests alternative competition models (see discussion). Furthermore, the appearance of ectopic eye cells in *β-cat1* RNAi animals, and the severe disorganization of the eyes of *β-cat1*/*β-cat4* RNAi planarians also suggest that *β-cat1* could exert additional autonomous roles, in pigment or neuronal cells, which influence the localization of photoreceptor cells.

Overall, our results indicate that the activity of β-cat1 in the eyes is not only controlled by the elements of the β-catenin destruction complex, like APC-1, but also by β-cat4, which exerts a negative regulatory effect on a process that is β-cat1 and APC dependent.

### *Smed-TCF-2* mediates *β-cat4* and *β-cat1* activity during photoreceptor differentiation

**Fig 4:**
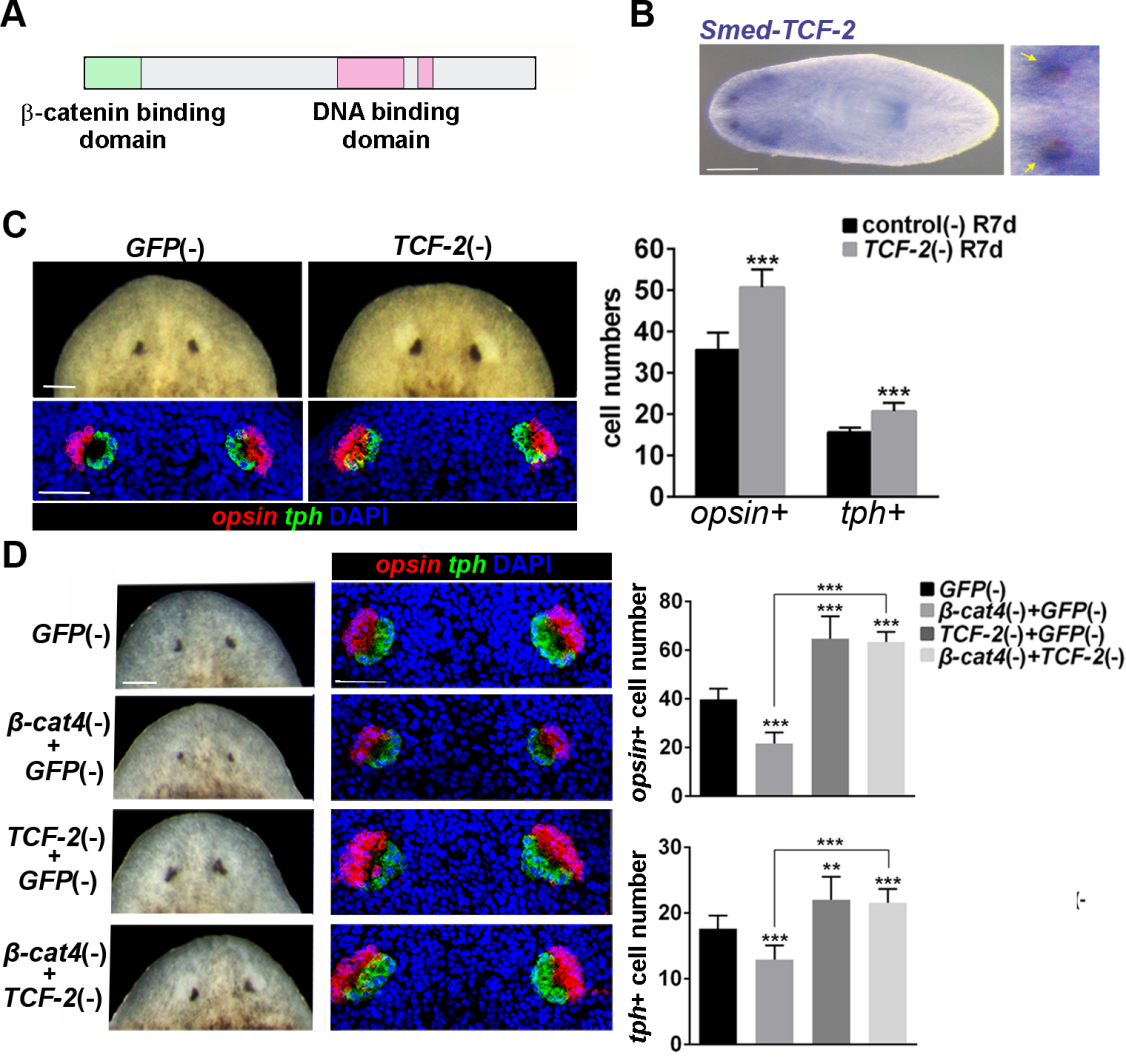
*TCF-2* mediates *β-cat1* and *β-cat4* signaling. **(A)** TCF-2 conserves the characteristic domains of *β-catenin* and DNA binding (HMG domain). **(B)** Expression of TCF-2 in the CNS and in the photoreceptors (yellow arrows in the magnification) **(C)** Live images and FISH of *opsin* (red) and *tph* (green) of *TCF-2* (RNAi) and *GFP* (RNAi) planarians at 7 days of regeneration, with the respective quantification of the *opsin+* and *tph+* cells per eye. *opsin+* cells in control R7d, 35.60±4.17 (SD; n = 10 eyes); *TCF-2* (RNAi) R7d, 50.8±4.21 (SD; n = 10 eyes). *tph+* cells in control R7d, 15.7±1.06 (SD; n = 10 eyes); *TCF-2* (RNAi) R7d, 20.70±2.06 (SD; n = 10 eyes). ***p<0.001 (t test). Anterior is to the top. **(D)** Double knockdown assay of *β-cat4* (RNAi) and *TCF-2* (RNAi). Live images and FISH of *opsin* (red) and *tph* (green) to show planarian regenerated eyes after the indicated RNAi treatment. The respective quantification of *opsin*+ and *tph*+ cells is shown. *opsin*+ cells in *GFP* (RNAi), 39.70±4.47 (SD; n = 10 eyes); *β-cat4*;*GFP* (RNAi), 21.60±4.48 (SD; n = 10 eyes); *TCF-2*;*GFP* (RNAi), 64.64±8.68 (SD; n = 10 eyes); *APC-1*;*β-cat4* (RNAi), 63.50±4.32 (SD; n = 12 eyes). *tph*+ cells in *GFP* (RNAi), 17.60±2.01 (SD; n = 10 eyes); *β-cat4*;*GFP* (RNAi), 12.89±2.15 (SD; n = 10 eyes); *TCF-2*;*GFP* (RNAi), 22.00±3.49 (SD; n = 10 eyes); *APC-1*;*β-cat4* (RNAi), 21.58±2.27 (SD; n = 12 eyes). **p<0.01, ***p<0.001 (t test). Scale bars, 250 μm (B), 50 μm (C, D).

To gain further insight into the mechanism through which planarian β-catenins specify photoreceptor differentiation, we searched for the TCF transcription factor that acts as a target. Although most invertebrate genomes contain a single TCF/LEF ortholog, we identified three TCF orthologs (Smed-TCF-1 to -3) in the *S*. *mediterranea* transcriptome database Planmine [58] (S8A Fig). The corresponding homologs were found in five more planarian species in the same database (S8A Fig). The phylogenetic analysis suggests that the duplications found in planarians arise from Platyhelmintes and are independent of the vertebrate TCF expansion (S8A Fig). Protein sequence analysis of the three *S*. *mediterranea* TCFs demonstrated that TCF-2 is the only *S*. *mediterranea* TCF that conserves all functional domains required to bind to β-catenin, Groucho and DNA (Fig 4A and S9 Fig) [59]. TCF-1 has lost the β-catenin binding domain and TCF-3 does not conserve the Groucho binding sites (S9 Fig). Analysis of their expression pattern in planarians showed that *TCF-1* was expressed specifically in the planarian brain, as previously reported [60] (S8B Fig). *TCF-2* and *-3* are also mainly expressed in the CNS and, importantly, *TCF-2* is found in photoreceptors (Fig 4B), strongly resembling the *β-cat4* expression pattern. Thus, TCF-2 is a candidate to function as a β-cat1 and β-cat4 target during photoreceptor development.

The eyes of *TCF-2* RNAi animals were analyzed to understand its possible function. The efficiency of the inhibition was analyzed by qPCR (S8C Fig). Analysis of *TCF-2* RNAi regenerating animals revealed that their eyes were bigger than in controls (Fig 4C and S8D Fig). The brain of *TCF-2* RNAi animals appeared normal (S8E Fig), which suggests that the larger eye phenotype is eye specific. Quantification of the different eye populations demonstrated that *TCF-2* silencing results in an increased number of photoreceptor cells (Fig 4C and S8D Fig). The number of pigment cells also increased (Fig 4C and S8D Fig), probably due to the cellular relationship between the two compartments, as shown earlier in *β-cat4* RNAi planarians. The *TCF-2* (RNAi) phenotype in the eyes phenocopies the *β-cat1* (RNAi) phenotype with respect to the number of photoreceptor cells and is consistent with the hypothesis that Wnt/β-cat1 signal inhibition is required for correct planarian photoreceptor specification. To note, *TCF-2* RNAi animals showed bigger eyes but not the appearance of ectopic eye cells, suggesting that this defect is caused in a *TCF-2* independent manner.

**Fig 5:**
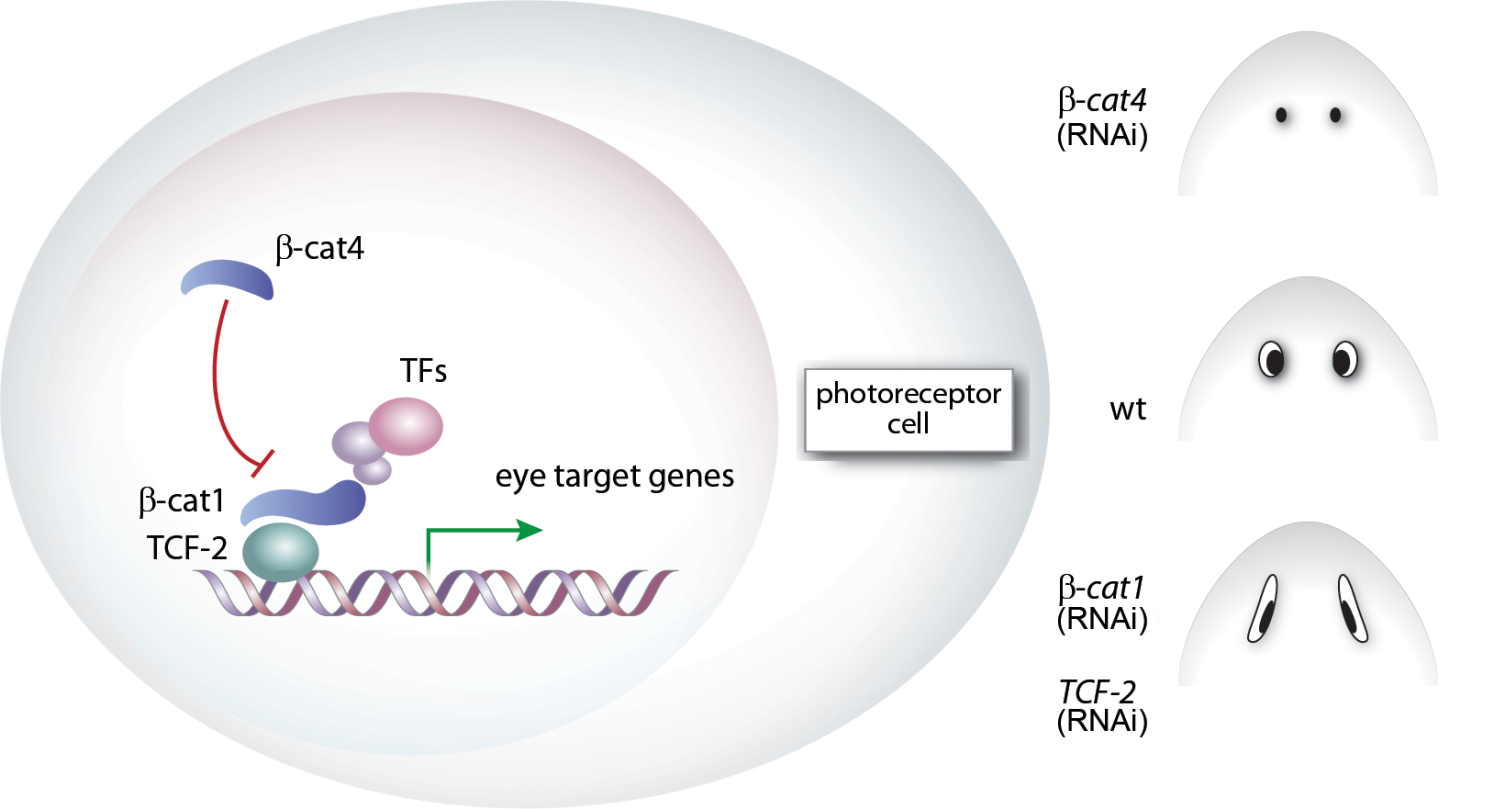
Proposed Model for Wnt/β-catenin activity modulation in planarian eyes. During planarian eye regeneration or maintenance, β-cat4 inhibits β-cat1/TCF-2 activity in photoreceptor cells, modulating Wnt signaling to an appropriate level to ensure correct differentiation of photoreceptors.

To analyze whether *β-cat4* function in photoreceptor specification also depends on *TCF-2*, we performed a double RNAi assay to inhibit *β-cat4* and *TCF-2* simultaneously during planarian regeneration. The efficiency of the inhibition was tested by qPCR (S8F Fig). As expected, *β-cat4* (RNAi) planarians had smaller eyes with a reduced number of photoreceptor cells, whereas *TCF-2* (RNAi) resulted in larger eyes with an increased number of photoreceptor cells. Remarkably, the size of the eyes in double *β-cat4* and *TCF-2* RNAi animals, and the number of photoreceptor and pigment cells, resembled the phenotype observed with *TCF-2* (RNAi) alone (Fig 4D). This observation is consistent with a role for TCF-2 as the transcription factor downstream of β-cat4. Above all, the data suggests that β-cat4, which lacks the C-terminal transactivation domain, modulates the β-cat1/TCF-2-mediated signal for the correct differentiation of photoreceptor cells (Fig 5).

### Plakoglobin and Neural Arm act as β-catenin and Arm inhibitors, respectively, in vitro

To determine whether the existence of inhibitory β-catenins could be an evolutionary conserved mechanism for the regulation of Wnt signaling, we investigated the existence of C-terminally truncated β-catenins in other organisms. The existence of *β-catenin* paralogs is not exclusive to planarians. The *β-catenin* family has undergone a vertebrate-specific subphylum duplication (*β-catenin* and *plakoglobin*) [61], two nematode-specific phylum duplications (4 *β-catenins* in *C*. *elegans*) [41], and multiple species-specific duplications in the Arthropoda phylum [61] (S10 Fig). Phylogenetic analysis of the *β-catenin* family members of different Lophotrocozoa species shows the existence of a unique bi-functional *β-catenin* in all of them except for Platyhelmintes (S10 Fig). The analysis suggests that *β-catenin* underwent two phylum-specific duplications in Platyhelmintes to generate *β-cat1*, *2* and *3/4* classes, and that in Triclads a third duplication produced the *β-catenin3* and 4 orthologs (S10 Fig). Furthermore, a genus-specific duplication occurred in the *β-cat3* class (S10 Fig). Thus, a *β-cat3*/4 ortholog that retains the signaling domains but not the C-terminal transactivation domain (S11 Fig) exists in all Plathyhelminthes.

**Fig 6:**
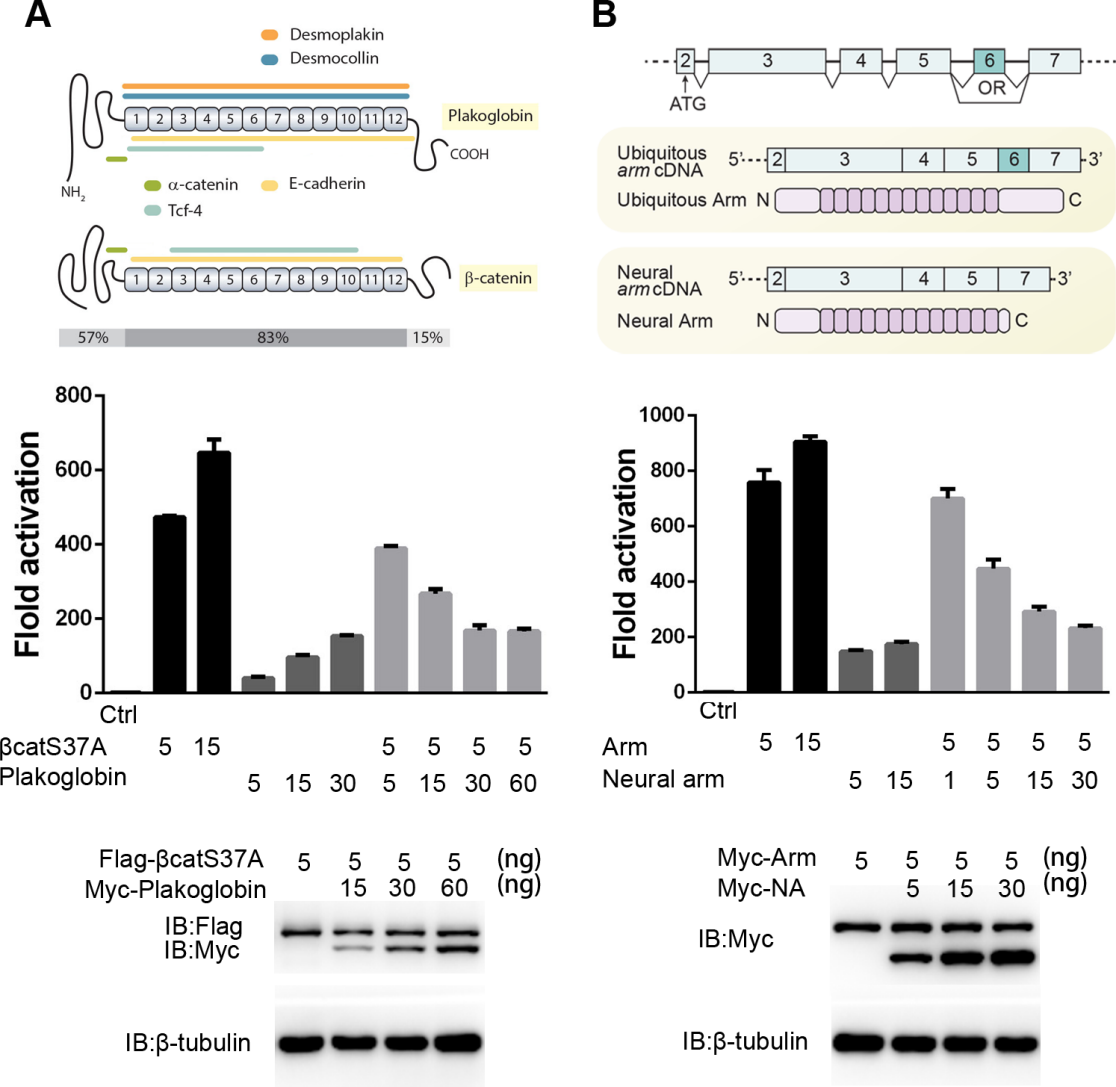
Plakoglobin and Neural Arm inhibit Wnt/β-catenin activity ‘in vitro’. **(A)** Upper panel, schematic representation of Plakoglobin or β-catenin proteins, showing the protein interacting domains and the degree of homology of the N-terminal, central Arm repeat and C-terminal region between both proteins. Lower panel, activation of TOPflash reporter signal by β-catenin (S37A) and Plakoglobin in HEK293T cells. Plakoglobin showed limited capacity to activate reporter signal, and inhibited β-catenin (S37A) activity in a dose-dependent manner. **(B)** Upper panel, schematic representation of the exon composition of Arm and Neural Arm (in which exon 6 is skipped). Lower panel, activation of TOPflash reporter signal by Armadillo and Neural Arm in HEK293T cells. Neural Arm showed limited capacity to activate reporter signal and inhibited Arm activity in a dose dependent manner.

Taking into account the number of β-catenin duplications found across evolution, we hypothesized that the existence of an inhibitory β-catenin to regulate β-catenin-dependent Wnt signaling could be a common mechanism throughout evolution as a result of convergent evolution. A protein sequence analysis of different *β-catenin* orthologs found across the animal phyla shows the shortening of the C-terminal end in several cases, for instance in one of the two β-catenins found in the sponge *A*. *quensatlantica* or the vertebrate β-catenin duplication Plakoglobin (S11 Fig). Although Plakoglobin shows high protein sequence conservation with β-catenin in the central armadillo repeats, and shares the binding domains for α-catenin, Cadherins and TCF, it has very low amino acid sequence conservation in the C-terminal transcriptional transactivation domain (15%) (Fig 6A) [62]. Accordingly, it has been shown that Plakoglobin has limited transactivation ability compared to β-catenin [63]. Furthermore, although a unique *β-catenin* is found in the genome of *D*. *melanogaster (Armadillo*, *Arm)*, a C-terminally truncated *Arm* named *Neural Armadillo* (*NArm*) has been reported to occur through alternative splicing [64] (Fig 6B and S11 Fig). It is known that *NArm* is expressed in the brain from larval stage [64] but no functional studies have been reported. To analyze whether vertebrate Plakoglobin or *Drosophila* NArm could have an inhibitory function, we designed *in vitro* Super-TOPflash experiments. Results showed that while β-catenin (S37A), a stabilized β-catenin that cannot be captured by the cytoplasmic destruction complex, could highly activate Wnt signaling, Plakoglobin activity was very low (Fig 6A). Interestingly, Plakoglobin co-transfection with β-catenin (S37A) decreased the reporter signal in a dose-dependent manner (Fig 6A). Similarly, activation of Wnt signaling by NArm was very weak compared to that induced by Arm. Importantly, co-transfection of both plasmids showed that NArm suppresses the Arm induced TCF-reporter activity in dosage dependent manner (Fig 6B).

Our results demonstrate that Plakoglobin and NArm inhibit the Wnt signal activated by β-catenin/Arm through a TCF transcription factor. This result, together with the presence of β-catenin paralogs in several species, indicates that the existence of an inhibitory β-catenin could be a conserved mechanism to fine tune β-catenin-dependent Wnt signaling. The phylogenetic relationship between the β-catenin family members and the existence of splice variants indicates that the inhibitory form of β-catenin would not have evolved from a common ancestor but appeared during evolution as a product of unrelated events (species- or phylum-specific genome duplications or alternative splicing), thus representing an example of convergent evolution.

## Discussion

Here we show a new role for the Wnt/β-catenin pathway in planarian eyes and demonstrate the existence of a novel mechanism to regulate its activity in a context-specific manner. We have identified *β-cat4*, a C-terminally truncated *β-catenin* generated by gene duplication within the planarian group, and we demonstrate that it modulates β-cat1 activity during eye photoreceptor cells specification but not during axial patterning. Wnt/β-catenin activity is modulated through a novel mechanism in which β-cat4, a truncated form of β-catenin that lacks the transactivation domain, exerts a negative regulatory effect on a β-cat1/TCF-2 dependent process. The existence of this new Wnt/β-catenin activity regulatory mechanism would ensure the appropriate fine-tuning of nuclear β-catenin/TCF activity in specific cell types. Genome searches and *in vitro* assays indicate that use of an inhibitory form of β-catenin could be a conserved mechanism to regulate β-catenin activity in specific contexts.

### The inhibitory role of the C-terminally truncated planarian β-catenin4 represents a novel mechanism for modulation of nuclear β-catenin activity.

The multiple roles and complexity of the Wnt/β-catenin signaling necessitate regulation at different levels. Extracellularly, secreted proteins interact with Wnts or their receptors (e.g. DKK1, SFRP, WIF1, notum) to inhibit the ligand-receptor interaction [65, 66], and in the cytoplasm, regulation of the β-catenin destruction complex determines the amount of β-catenin that will escape phosphorylation and degradation and reach the nucleus [5]. However, the modulation of β-catenin activity once it reaches the nucleus remains poorly understood. We used planarians to approach this question since, although β-cat1 exerts multiple functions, it is primarily localized to the nucleus [31–33, 36, 44–46, 48], suggesting that mechanisms must be available to modulate β-cat1 nuclear activity.

Here we found two new β-catenins (β-cat3 and 4) in planarians, after the two β-catenins (β-cat1 and 2) already described [40], which 1) showed a C-terminal truncated transactivation domain, while conserving the TCF binding amino acid residues, and 2) were mainly expressed in the nervous tissues. Those findings lead to hypothesize that β-cat3 and 4 could be acting as inhibitors of the canonical β-cat1 in the nucleus and in a tissue-specific manner. Indeed, we could demonstrate that *β-cat4* is required for normal specification of photoreceptors from the common eye stem cell and that its function relies on the inhibition of β-cat1 activity. Importantly, *β-cat4* has no role in A-P axial polarity, in contrast to *β-cat1*.

Our RNAi experiments show that inhibition of *β-cat4* or *APC*, which in planarians is demonstrated to increase β-cat1 activity [31, 34, 45], produces a decrease in the number of photoreceptor cells, whereas inhibition of *β-cat1* produces the opposite phenotype, indicating that β-cat4 exerts a negative regulatory effect on a β-cat1-dependent transcriptional activity. The finding that inhibition of *TCF-2*, the TCF factor identified in the present study, leads to an increase in the number of photoreceptor cells, as observed after *β-cat1* RNAi, and that RNAi inhibition of *β-cat4* together with *TCF-2* leads to the same phenotype as *TCF-2* RNAi alone, indicates that TCF-2 is the downstream effector of the β-cat1 and β-cat4 action. Although we cannot rule out the possibility that β-cat1 and β-cat4 could interact in the cytoplasm, since β-cat4 conserves the essential domains to interact with cytosolic β-catenin destruction elements, our results support the regulative interaction between β-cat1 and β-cat4 at nuclear level. Thus, both β-cat1 and β-cat4 are found in the nucleus of photoreceptor cells (although β-cat1 expression analysis was performed in a *S*. *mediterranea* sister species due to technical limitations [45]). Furthermore, although we have not directly demonstrated the binding of β-cat1 or β-cat4 to TCF-2, we demonstrate that both planarian β-catenins conserve the TCF binding sites and bind to xTCF-1 in co-immunoprecipitation experiments.

**Fig 7:**
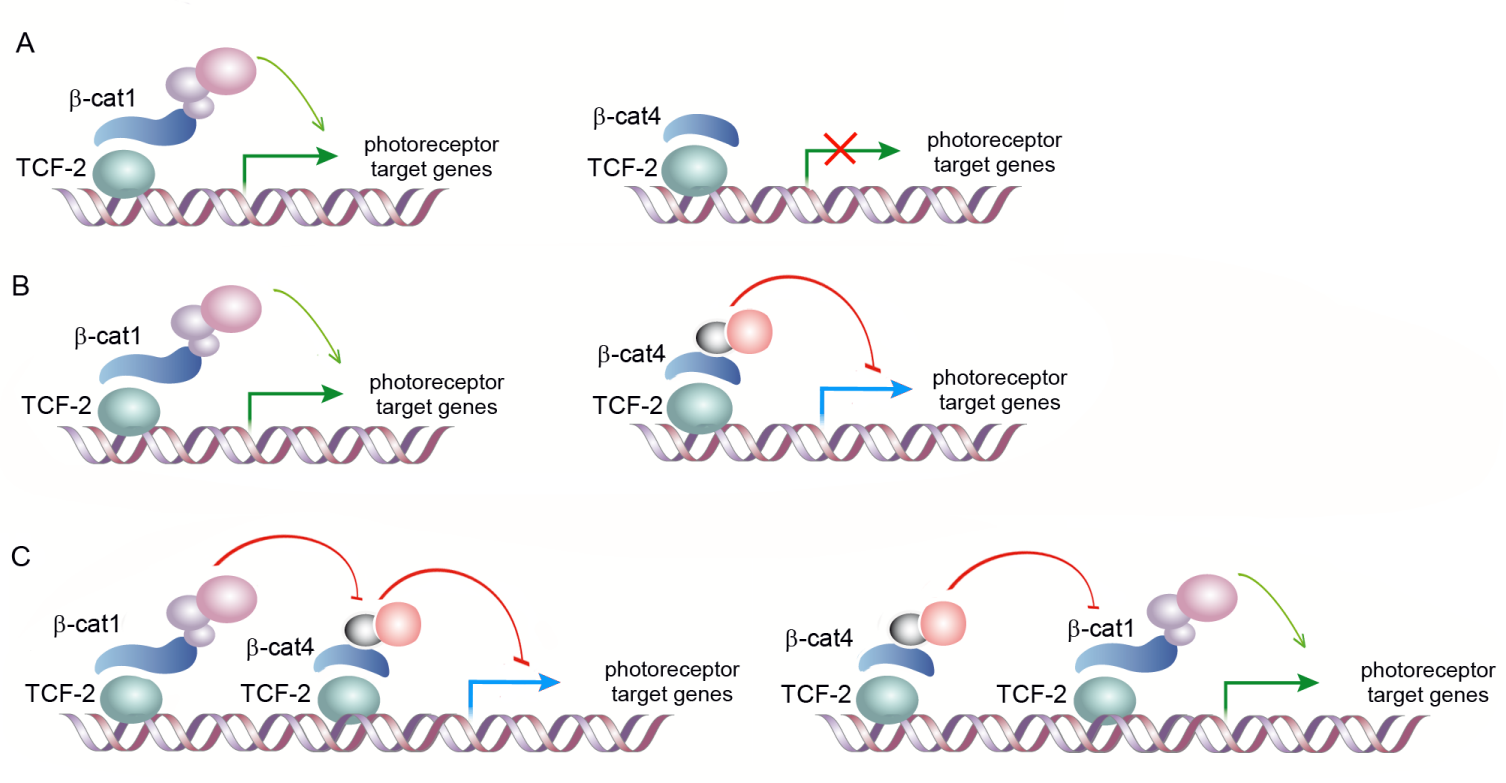
Possible Models for β-cat1 and β-cat4 transcriptional activity in planarians. **(A)** β-cat4 could act as a dominant negative form of β-catenin, which is able to bind to TCF-2 but not to activate transcription, due to the missing C-terminal domain. **(B)** β-cat4 could be able to directly repress transcriptional targets through binding to TCF-2. The balance between β-cat1/TCF-2 transcriptional activation and β-cat4/TCF-2 transcriptional repression would determine the amount of photoreceptor cells. **(C)** Both β-cat1/TCF-2 and β-cat4/TCF-2 complexes could bind to different TCF responsive elements in the same promoter. The proper transcriptional regulation of photoreceptor targets is achieved by the combinatory action of both complexes.

Overall, our results in planaria indicate that β-cat1 and β-cat4 regulate in an opposite manner the transcription of genes required for photoreceptor specification, and that this action depends on the TCF-2 transcription factor. According to the presented data, three main scenarios could be considered (Fig 7). In the first one, β-cat4 could act as a dominant negative form of β-catenin, which is able to bind to TCF-2 but not to activate transcription, due to the missing C-terminal domain (Fig 7A). The RNAi phenotypes of *β-cat1*, *β-cat4*, and *TCF-2*, agree with this possibility. However, the finding that the double *β-cat1/ β-cat4* inhibition produces a decrease in the number of photoreceptors cannot be directly explained under this supposal. In the second scenario, β-cat4 could be able to directly repress transcriptional targets through binding to TCF-2 (Fig 7B). In *Drosophila*, it has been shown that Arm and TCF can directly repress transcriptional targets and that this repressive activity of Arm does not require the C-terminal domain [67, 68]. Thus, the balance between β-cat1/TCF-2 transcriptional activation and β-cat4/TCF-2 transcriptional repression would determine the amount of photoreceptor cells. The finding that double inhibition of *β-cat1* and *β-cat4* does not phenocopy the *β-cat1* RNAi phenotype, favor this hypothesis. However, under this scenario the *β-cat1*/*β-cat4* RNAi should produce a similar phenotype to the one produced after *TCF-2* inhibition, which is not the observed. A third possibility would be that the proper transcriptional regulation of photoreceptor targets is achieved by the combinatory action of both β-cat1/TCF-2 and β-cat4/TCF-2 complexes when bind to different TCF responsive elements (Fig 7C). In the future, the analysis of specific downstream targets of β-cat1, β-cat4 and TCF-2, and the possibility of performing tissue specific RNAi and rescue experiments would help to clarify the specific mechanism of β-cat1 and β-cat4 activity.

Altogether, our data supports the existence in planarians of a novel level of Wnt/β-catenin signaling modulation in the nucleus through the action of β-catenins with different transactivation capacity. The regulatory interaction between those different β-catenins and TCFs would result in the modulation of the transcriptional rates of downstream Wnt target genes and Wnt-responsive activities.

Regulation of β-catenin/TCF transcriptional activity is achieved through nuclear factors that regulate β-catenin binding properties (e.g. Chibby and ICAT) [20, 22–27], or regulation of TCF transcriptional and binding properties [20, 22–27]. Accordingly, the existence of TCFs that lack the β-catenin-binding domain and act as endogenous dominant negative forms [59], and also post-translational modifications of TCFs that modulate their activity have been extensively reported [69–74]. The C-terminal truncated inhibitory β-cat4 found in this study represents a novel mechanism to modulate β-catenin/TCF activity that also targets to TCF. As reported for the endogenous dominant negative forms of TCFs, in which alternative splicing and alternative promoters leads to the expansion of the isoforms [59], this level of regulation would allow the refinement of Wnt signaling in time and space.

### A C-terminal truncated form of β-catenin could be an evolutionarily conserved mechanism to achieve the multiple context-specific roles of Wnt/β-catenin signaling

Here we have found a new planarian β-catenin (β-cat4) which represents a novel mechanism to fine tune the activity of nuclear β-catenin in a context-specific manner. Not surprisingly, this mechanism acts in neural tissues, where *β-cat3* and *4* are mainly expressed, the complexity of which requires a more sophisticated regulation. Phylogenetic analysis of the β-catenin family in platyhelminthes supports the hypothesis that a *β-cat1*, *β-cat2* and *β-cat3/4* ortholog was present in their common ancestor. Although the expression pattern and functional analysis of the *β-cat3/4* ortholog in other platyhelminth species should be done, our data predicts that the *β-cat3/4* ortholog would modulate β-cat1 activity in Platyhelminthes.

Importantly, although the presence of a unique *β-catenin* with dual adhesion and signaling functions has been proposed to be present in the last common animal ancestor [75], genomic duplications in the *β-catenin* family are more common than previously thought. In vertebrates, *plakoglobin* is a genomic duplication of *β-catenin* that has suffered a functional specialization, since it is predominantly involved in desmosomes [76, 77]. However, Plakoglobin shows limited capacity to activate Wnt signaling [63, 78] and, importantly, its nuclear accumulation down-regulates β-catenin activity in a dose-dependent manner, as demonstrated by our TOPflash reporter assays (Fig 6A). Thus, Plakoglobin could be acting as a negative regulator of β-catenin in vertebrates. Since Plakoglobin shows much lower transactivation properties than β-catenin, its presence results in a decrease of the transcriptional activation promoted by β-catenin. The real existence and the importance of this regulatory mechanism is further supported by recent studies demonstrating that an increase of Plakoglobin nuclear translocation is associated with diseases such as arrhythmogenic right ventricular cardiomyopathy or head neck cancer and leads to a suppression of the β-catenin mediated TCF/LEF transcriptional activity [79, 80]. Taking into account that the C-terminal part of Plakoglobin is the one showing less conservation with respect to β-catenin (Fig 6A), the inhibitory function could be associated with the lack of conservation of the transactivation domain, as described in the present study for planarian β-cat4.

In invertebrates, the *β-catenin* family has undergone several species- and phylum-specific duplications [81]. The nematode *C*. *elegans* has four β-catenins with different roles in cell adhesion and nuclear signaling [42], and several insects have two β-catenins, which have also undergone partial subfunctionalization between the cell adhesion and the centrosome separation functions [81]. Importantly, although in *Drosophila* a unique *β-catenin* is found (*Arm*), an alternative splicing which occurs mainly in neural cells has been reported, which is known as *Neural Armadillo* (*NArm*) [81]. Interestingly, this alternative splicing deletes exon 6 and results in a C-terminal truncated Arm isoform that, according to our TOPflash reporter assay, exerts an inhibitory role of Arm in a dose dependent manner, as described for planarian *β-cat4* towards *β-cat1*. Although the role of NArm as modulator of Arm should be demonstrated ‘*in vivo*’, it should be stressed that the presence of the alternative splicing isoform deleting exon 6 is conserved across all insect homologs [81], supporting the evolutionarily pressure to maintain it and thus its biological implications. Overall, the present data supports the hypothesis that modulation of nuclear β-catenin transcriptional activity through the action of a C-terminally truncated β-catenin able to bind to TCF could be a conserved mechanism to regulate the Wnt/β-catenin pathway. Interestingly, the C-terminally truncated *β-catenin* of different species did not arise from any homolog of a common ancestor but originated in different animal groups from different mechanisms of gene diversification, as gene duplication or alternative splicing. Thus, the presence of a β-catenin with limited transactivation activity, which could compete with the canonical β-catenin, in different species represents an example of convergent evolution and supports its importance as a regulatory mechanism.

The existence of inhibitory β-catenins and its expression in specific cell types provides a new answer to the important question of how the same transcription factor elicits so distinct responses. It is assumed that the different transcriptomic/proteomic context of each cell could result in differential activation of β-catenin target genes. However, few tissue-specific Wnt/β-catenin regulators have been found, leading to the idea that not only Wnt/β-catenin regulators but the interplay with the other conserved signaling pathways as BMP would be essential for their complex regulation [82]. Our results provide evidences that the existence of inhibitory β-catenins is not only a new mechanism to regulate Wnt signaling but to modulate it in specific cell types, for instances in specific subsets of neuronal types.

### Eye development and Wnt/β-catenin signaling

Our results demonstrate that *β-cat4* is a new essential element for photoreceptor cell specification in planarians. Planarian eyes are true cerebral eyes and their simplicity makes them an excellent system to study eye development. They are composed by two cell types: rhabdomeric photoreceptor neurons, which express evolutionarily conserved photoreceptor genes such as *otxA* or *opsin* [49, 55], and pigment cells, which express *Tryptophan hydroxylase (tph)*, an enzyme involved in the production of melanin, the pigment found in planarian eyes. Several studies demonstrate that both cell types arise from a common eye stem cell that express stem cell markers (*h2b*) and eye determinants as the transcription factors *ovo*, *eya* and *six1/2* [54, 55, 83]. Among them *ovo* is the only factor exclusively found in the eye cell and specifically essential for eye regeneration and maintenance [54]. The expression of *sp6-9* and *dlx* in the common eye progenitor determines the pigment fate, while *otxA* specifies photoreceptors [52, 55]. We demonstrate that *β-cat4* is a new factor that specifies the photoreceptor fate and that it is expressed in the stem cell precursors (*h2b+/ovo+*), a fact that has not been yet demonstrated for *otxA*. Inhibition of *β-cat4* results in a decrease in number of photoreceptor progenitor and differentiated cells from the earliest time points analyzed (3–4 days of regeneration), while the number of progenitors and differentiated cells of the pigment lineage only decrease several days after. This observation agrees with the already described mutual dependence of both cell populations in planarians [53]. Thus, although *β-cat4* is specifically required for photoreceptor determination, according to its expression in the photoreceptor lineage, the reduced number of photoreceptor cells would lead to the subsequent decrease of the pigment lineage. Whether the origin of this mutual dependence is at the level of the common stem cell progenitors, or at the level of the differentiated cells in the optic cup, remains to be studied. Although not much is known about the mutual dependence between photoreceptor and pigment cells in vertebrates, some studies suggest that it also exists [84].

To date, the main focus on the role of the Wnt/β-catenin signaling pathway in planarians has been in its essential role for axial patterning and posterior identity specification [31–33]. Our results highlight the importance of the regulation of the Wnt/β-catenin pathway in a different context, namely during planarian photoreceptors regeneration and maintenance. The finding is not surprising, since appropriate Wnt/β-catenin signaling levels are required for retina progenitor differentiation in several animal models [85–90]. For example, during chicken embryo development, β-catenin overexpression in the central neural retina inhibits the differentiation of retinal neurons, while loss of β-catenin leads to neural retina enlargement [86]. In zebrafish the ectopic expression of the Wnt antagonists DKK1 or SFRP1 causes expansion of the embryonic retina [91, 92], whereas activation of the Wnt/β-catenin pathway leads to lack of expression of eye markers [93]. Here we show that in planarians *β-cat1* inhibition results in larger eyes, while *APC-1* silencing causes a reduction of photoreceptor cell number. The simplicity and approachability of planarian eyes has allowed the identification of a new mechanism to fine regulate Wnt/β-catenin pathway activity in the eyes. Thus, the truncated β-cat4 that competes with the canonical β-cat1 for binding to TCF-2 allows the required fine-tuning of the Wnt/β-catenin pathway in planarian photoreceptor cells. Since the *in vitro* experiments showed that β-cat4 could bind to E-cadherin and α-catenin, a role of β-cat4 in cell adhesion should be considered. However, β-cat4 protein was only found in the nucleus of photoreceptors (Fig 3A), and *β-cat4* RNAi eyes appeared properly patterned with no apparent defects in cell adhesion.

The new described mechanism to fine-tune the Wnt/β-catenin signal in specific cell types implies the existence of Wnt elements specifically expressed in photoreceptor and not in pigment cells. The finding that the phenotype of *β-cat1* RNAi animals, which show ectopic differentiation of eye cells, differs from the *TCF-2* RNAi, which show bigger eyes but properly patterned, indicates that β-cat1 could exert a TCF-2 independent role during eye regeneration. The cause of this defect could be a non-autonomous role of β-cat1, which is broadly expressed in planarians [45], or a role in pigment cells. This finding emphasizes the requirement of a cell-specific regulation of the Wnt/β-catenin signal during development. It will be interesting to study whether in other animal species eye-population specific Wnt elements (or isoforms) are found and to investigate if this novel mechanism of β-catenin regulation also takes place during eye development.

## Conclusions

Our finding that planarian β-cat4 functions as a modulator of β-cat1 activity in the nucleus by regulating TCF-2 transcriptional activity has two deep implications: 1) considering its specific role in modulating β-cat1 activity during planarian photoreceptor specification, and not in other Wnt/β-catenin dependent processes such as axial patterning, it appears as a tissue-specific manner to fine-tune nuclear β-catenin activity; and 2) the finding of C-terminal truncated/non-conserved β-catenin forms in other species (NArm or Plakoglobin), which inhibit β-catenin activity in TOPflash experiments, suggests that an inhibitory β-catenin could be an evolutionarily conserved mechanism to regulate the Wnt/β-catenin signaling. The identification of a novel mechanism of Wnt/β-catenin signaling regulation has important implications in view of the complex and essential roles of this pathway in development and diseases. Thus, the present research represents a starting point to design further studies: 1) to demonstrate whether the C-terminal truncated/non-conserved β-catenins found in *Drosophila* (NArm) and vertebrates (Plakoglobin) can compete for Arm or β-catenin binding, respectively, to the TCF co-factor ‘in vivo’ and assess whether this mechanism provides a tissue-specific manner to modulate β-catenin/Arm once reaches the nucleus; 2) to study the existence and function of possible β-catenin isoforms present in other animal species, as a product of gene duplication or alternative splicing, and understand their real contribution to the regulation of the Wnt/β-catenin signal; and 3) to test whether new drugs mimicking the inhibitory action of β-cat4 could be used to fine tune the activity of nuclear β-catenin in human diseases.

## Materials and methods

### Planarians

The planarians used in this study belong to an asexual clonal strain of *S*. *mediterranea* BCN-10 biotype. The animals were maintained at 20°C in PAM (1X) water [94]. Animals were fed with organic veal liver and starved for at least a week before all experiments. A sexual strain of *S*. *polychroa* collected from Sant Celoni (Barcelona, Spain) was used in Fig 3A.

### Gene identification and phylogenetic analysis

*β-Catenin* sequences from Planarian species were identified in the Planmine database [58]. *β-Catenin* sequences from *Echinococcus multilocularis* and *Kronborgia cf*. *amphipodicola* were found in the available databases [95, 96]. The rest of the sequences were found in the NCBI. β-Catenin protein sequences from different species were aligned using the MAFFT server (http://mafft.cbrc.jp/alignment/server/). Neighbor joining distance-based analyses were conducted using MEGA version 6 [97], and the support given by bootstrap percentiles of 1000 replicates. BioEdit was used to edit the protein alignments.

### Luciferase reporter assay

HEK293T cells were cultured in Dulbecco’s modified Eagle’s medium (DMEM) supplemented with 10% fetal calf serum. To obtain pCS2+-6Myc-Smed-β-catenin3 and 4, the full length Smed-β-catenin3 and 4 was amplified by PCR and inserted into pCS2+-6Myc vector at StuI/XbaI sites. HEK293T cells were seeded in 96-well plates and transfected in triplicates with pCS2+-6Myc-Smed-β-catenin1/2/3/4 plasmids, together with Super-TOPflash and pRL-TK as the internal control. Firefly and Renilla luciferase activities were measured 36h after transfection using the Dual-Luciferase assay kit (Promega). TOPflash luciferase activity was normalized to that of Renilla. 15 ng of Super-TOPflash and 0.5 ng of pRL-TK reporter plasmids were added per well. pCS2+ empty vector was used to adjust the total DNA amount to 150 ng/well. All experiments were repeated at least three times.

### Immunofluorescence in cell cultures

HeLa cells were cultured in DMEM with 10% fetal calf serum and grown on glass coverslips. Thirty-six hours post transfection of pCS2+-6Myc-Smed-β-catenin3/4 and pCS2+-HA-mAxin, cells were fixed with4% paraformaldehyde/PBS for 20 min, permeabilized with 0.2% Triton X-100/PBS for 10 min and then blocked with 3% BSA/PBS for 30 min before primary antibodies were applied. A PBS rinse for 5 min between each step was performed. The primary antibodies anti-Myc (mouse, Santa Cruz) and anti-HA (rabbit, Santa Cruz) were incubated for 1h at RT diluted in 1%BSA, 0.1%Tween 20/PBS at 1:100. After washing 5min X 3 times in 3%BSA, 1%TritonX-100/PBS, the fluorophore-conjugated secondary antibodies diluted at 1:400 in 3%BSA/PBS were incubated for 1h. Donkey anti mouse–Alexa Fluor 568 (Molecular Probes) was used to visualize β-catenin3/4, and goat anti rabbit–Alexa Fluor 488 (Molecular Probes) to visualize Axin. Cell nuclei were visualized with DAPI staining. Cells were mounted in Prolong Gold antifade reagent (Thermo Fisher Scientific) and stored at 4°C before imaging. Images were recorded using a Zeiss LSM710 confocal microscope.

### Co-immunoprecipitation assay and western blot

HEK293T cells were seeded into 6-well plates and the following plasmids transfected the following day: pCS2+-6Myc-Smed-β-catenin1/2/3/4, pCS2+-flag-xTCF1, pcDNA3.1-flag-β-Trcp, pcDNA3.1-flag-E-cadherin and pcDNA3.1-flag-α-catenin. Forty-eight hours after transfection, cells were lysed and sonicated in 400 μl of lysis buffer/well (50 mM Tris-HCl, pH 7.4, 300 mM NaCl, 1 mM EDTA, pH 8.0, 1% NP-40) containing protease inhibitor mixture (Roche Applied Science) at 4°C. After centrifugation at 14000 rpm, 4°C for 15min, 40 μl of supernatant was mixed with 10 μl 5x SDS-loading buffer and treated at 95°C for 5min. The remaining supernatant for each well was incubated with 10 μl of FLAG-M2 beads (Sigma) at 4°C for 6h. The beads were then washed three times with lysis buffer at 4°C for 10min each, and bound proteins were eluted with 40 μl of 2x SDS loading buffer at 95°C for 5min. Immunoprecipitates and total lysates were separated by SDS-PAGE and analyzed by immunoblot with anti-Myc or anti-Flag specific antibodies.

### *Xenopus* embryo assays

*Xenopus* embryos were cultured under standard conditions. mRNA was synthesized using a mMESSAGE mMACHINE SP6 kit (Ambion, Austin, TX) according to the manufacturer’s instructions. Synthetic mRNAs were microinjected into embryos cultured in 2% Ficoll 400 in 0.3× MMR (1× MMR: 100 mM NaCl, 2 mM KCl, 2 mM CaCl2, 1 mM MgCl2) at 8-cell stage, and fixed at stage 20.

### *In situ* hybridization in whole-mount planarians

Colorimetric whole-mount *in situ* hybridization (WISH) and fluorescent *in situ* hybridization (FISH) were performed as elsewhere described [98, 99]. The following DIG- (Roche), FITC- (Roche), or DNP- (Perkin Elmer) labeled riboprobes were synthesized using an *in vitro* transcription kit (Roche): *Smed-β-catenin1/3/4*, *Smed-TCF1/2/3*; *Smed-opsin*, *Smed-otxA*, *Smed-sp6/9* [52]; *Smed-tph* [100]; *Smed-ovo* [54]; *Smed-h2b* [101]; *Smed-th* (tyrosine hydroxylase), *Smed-tbh* (tryptophan hydroxylase) [102], *Smed-pc2* (*prohormone convertase 2*) [103]. Primers used for their synthesis are indicated (S1 Table). Riboprobes were finally diluted to 250 ng/μL in pre-hybridization solution, stored at -20°C, and were used at 1:500 in hybridization solution, except for *sp6/9* (1:200). Samples were observed through Leica MZ16F (Leica Microsystems, Mannhiem, BW, Germany), Zeiss Stemi SV6 stereomicroscopes and a Zeiss Axiophot microscope (Zeiss, Jena, TH, Germany); images were captured with a ProgRes C3 camera from Jenoptik (Jena, TH, Germany), sCMEX 3.0 camera (Euromex, Arnhem, The Netherlands) and Leica DFC300FX camera (Leica Microsystems, Heerbrugg, CH, Switzerland). Confocal laser scanning microscopy was performed with a Leica TCS-SP2 (Leica Lasertchnik, Heidelberg, BW, Germany) adapted for an inverted microscope.

### Immunohistochemistry in planarians

After FISH, samples were rinsed with TNTx 5min, 50%PBSTx, 50%TNTx (0.1M Tris•HCl pH7.5, 0.15M NaCl, 0.3%TritonX-100) 10min, then PBSTx 10min. 1% BSA or 10% Goat serum were used as blocking reagent for 2 hours, followed by anti-*β-cat4* (1:200, diluted in 10% goat serum) or anti-VC-1 (1:15000, diluted in 1% BSA, kindly provided by Hidefumi Orii, Himeji Institute of Technology, Hyogo, Japan) antibody incubation. PBSTx (PBS with 3% TritonX-100) wash for 15min x3. Goat-anti-mouse-488 conjugated antibody (1:400, Molecular Probes) and Goat-anti-rabbit-HRP conjugated antibody (1:500, Pierce) were used as secondary antibody. HRP signal was developed with a tyramide signal amplification kit following manufacturer’s recommendations (Perkin Elmer). anti-β-cat1 immunohistochemistry was performed as previously described [45]. Nuclei were counterstained with DAPI (Sigma, 1:5000). The polyclonal anti-β-cat4 antibody was generated against 34 amino acids of the N-terminal part of the β-cat4 protein (indicated in S1 Fig) (GeneCust, Luxembourg).

### RNAi silencing

Double-stranded RNAs (dsRNA) for *Smed-β-catenin1/3/4*, *Smed-TCF2* and *Smed-APC-1* were synthesized and delivered as described elsewhere [49]. Primers used for their synthesis are indicated (S1 Table). Control animals were injected with dsRNA for GFP [53]. dsRNA was diluted to 1 μg/μl in water. Microinjections were performed as described elsewhere [49] following the standard protocol of a 32 nl injection of dsRNA on three consecutive days. On the next day planarians were amputated pre- and post-pharyngeally, and the head, trunk, and tail pieces were allowed to regenerate. When injecting *Smed-β-catenin3/4* the same procedure was performed during 2 weeks to improve the penetrance of the phenotype. For experiments in intact planarians, animals were injected 3 consecutive days per week for 5 weeks.

### Quantitative real-time PCR (qPCR)

Total RNA was extracted from a pool of 4 planarians each for every RNAi condition. Quantitative real-time PCR was performed as previously described [104], and data was normalized based on the expression of URA4 as internal control. All the experiments were performed using three biological replicates. The primers for qPCR are indicated in S1 Table. Statistical significance was measured by Student's T test by comparing values from each sample to their respective control sample.

### Phototactic assay

Phototactic assay was carried out as described previously [53]. The behavior analysis software SMART v.2.5.21 was used to quantify the numbers of worms in each of the three virtual subdivisions of a 60x30x10 mm transparent container filled with 10 ml planarian water after 2 minutes of positioning the worms at the indicated beginning point in the light zone.

## Supporting information

S1 Fig Protein sequence analysis of β-catenin homologs from different species. Protein regions and amino acids required for Wnt signaling are indicated in red. β-cat3 and 4 conserve the GSK3 phosphorylation sites in the N terminus, the hydrophobic pocket formed by Phe 253 and some of the surrounding residues, essential for Axin and TCF binding [105]. The red line underlies the C-terminal transactivation domain, which is necessary and sufficient for signaling through TCF factors [106, 107]. The C-terminal transactivation domain is lost in β-cat3 and 4 proteins. Amino acids required for cell-cell adhesion but also involved in TCF interaction, which are conserved in β-cat3 and 4 proteins, are labeled in blue: the armadillo repeats 4–9 in human β-catenin constitute a core interacting platform for cadherin and TCF binding, in which Lys312 and Lys435 form the critical salt bridges [108, 109]. The α-catenin binding sites [110, 111] appear conserved in β-cat4 but not in β-cat3. Armadillo repeats are underlined with a violet line. The conserved two last repeats, which are not present in β-cat3 and 4 proteins, are underlined by a dashed line. The Helix-C (orange square), a helix-α structure found C-terminal to the last Armadillo repeat and required for transcriptional co-activation [112], is also not conserved in β-cat3 and 4 proteins. The amino acids used to generate the anti β-cat4 antibody are underlined in yellow. Accession numbers of the sequences analyzed are: Hv-bcat, AAQ02885.1; Pd-bcat, ABQ85061.1; Hs-bcat, NP_001895.1; Sp-bcat, NP_001027543.1; Dm-Arm, NP_476666.1; Smed-bcat1, ABW79875.1; Smed-bcat2, ABW79874.1. The accession numbers of the new planarian β-catenins are: β-cat3, KY196224; and β-cat4, KY196225. Abbreviations: *Hv*, *Hidra vulgaris; Pd*, *P*. *dumerilii; Hs*, *H*. *spiens; Sp*, *Strongilocentrotus purpuratus; Dm*, *Drosophila melanogaster; Smed*, *S*. *mediterranea*.
(TIF)

S2 Fig β-cat3 and 4 inhibit β-cat1-dependent Wnt signaling *in vitro*. **(A-B)** Co-immunoprecipitation assays in 293T cells showed the ability of β-cat4 to interact with E-cadherin and α-catenin, while β-cat3 could only interact with E-cadherin. β-cat1 was used as a control since it is reported its interaction with E-cadherin [40]. (**C)** Axis duplication assays in *Xenopus* embryos. *β-cat1* mRNA injection induces a secondary axis in *Xenopus* embryos, as reported [32, 40], whereas no effects are observed after *β-cat3* or *4* mRNA injection. However co-injection of *β-cat3/4* together with *β-cat1* rescued the “double axis” phenotype. The percentages of embryos with the indicated phenotypes are shown (n = 50).
(TIF)

S3 Fig *β-cat3* and *4* expression pattern and their RNAi phenotype. **(A)** Both *β-cat3* and *β-cat4* were mainly expressed in the CNS after WISH and FISH (red). *β-cat4* was also expressed in photoreceptors (see magnification in the orange box). Analyzed planarians correspond to intact animals. (**B)** *β-cat3* and *β-cat4* expression pattern during regeneration of trunk fragments, which must regenerate the head and the tail. *β-cat3* and *β-cat4* were expressed in the newly formed brain (arrows in R3d) and *β-cat4* is also expressed in the regenerating eyes (see magnification in the orange box). **(C)** Phenotype of control and *β-cat3* and *4* RNAi animals after dsRNA injection and induced regeneration. No polarity defects were observed in *β-cat3* or *β-cat4* RNAi animals. No obvious affection in the brain was neither observed through analysis of brain markers, 3C11 (green), *Smed-th* (tyrosine hydroxylase) (red), and *Smed-tbh* (tryptophan hydroxylase) (green) [102]. Planarians shown were trunk fragments at 12 days of regeneration. **(D)** *β-cat1 RNAi* caused anteriorization of regenerating head fragments, as expected [31–33], while *β-cat4 RNAi* planarians show a normal regenerated posterior region, indicating that *β-cat4 RNAi* does not cause antero-posterior defects. **(E)** Relative expression levels of *β*-cat1/2/3/4 after *β-cat4 RNAi* by qRT-PCR. Values represent the means of three biological replicates. Error bars represent standard deviation. Data were analyzed by Student′s t-test. *p<0.05; **p<0.01; ***p<0.001. Scale bars = 250 μm (A, B, C, D) and 50 μm (magnification in A and B).
(TIF)

S4 Fig *β-cat4* (RNAi) produces a decrease of photoreceptor cells during planarian regeneration. Double FISH of *opsin* (red) and *tph* (green) in control and *β-cat4* (RNAi) animals, at 4, 7, and 14 days of regeneration. Scale bar = 50 μm.
(TIF)

S5 Fig *β-cat4* (RNAi) produces a decrease of photoreceptor cells during planarian homeostasis. **(A)** *β-cat4* (RNAi) planarians after 5 weeks injection show smaller eyes with small photoreceptor area. FISH of *opsin* (red) and *tph* (green) in control and *β-cat4* (RNAi) intact animals along the 5 weeks of *β-cat4* dsRNA injection and its quantification, showing a decrease mainly in photoreceptor cells. *opsin*+ cells in control h2w, 51.80±4.71 (SD; n = 10 eyes); *β-cat4* (RNAi) h2w, 30.75±5.20 (SD; n = 8 eyes); control h3w, 45.40±6.26 (SD; n = 10 eyes); *β-cat4* (RNAi) h3w, 25.50±2.62 (SD; n = 8 eyes); control h4w, 39.40±4.25 (SD; n = 10 eyes); *β-cat4* (RNAi) h4w, 18.70±1.64 (SD; n = 10 eyes); control h5w, 37.08±4.14 (SD; n = 10 eyes); *β-cat4* (RNAi) h5w, 12.50±1.35 (SD; n = 10 eyes). *tph*+ cells in control h2w, 18.60±1.90 (SD; n = 10 eyes); *β-cat4* (RNAi) h2w, 16.38±1.41 (SD; n = 8 eyes); control h3w, 18.80±2.00 (SD; n = 10 eyes); *β-cat4* (RNAi) h3w, 13.25±0.89 (SD; n = 8 eyes); control h4w, 17.40±1.43 (SD; n = 10 eyes); *β-cat4* (RNAi) h4w, 10.80±1.32 (SD; n = 10 eyes); control h5w, 16.00±3.10 (SD; n = 12 eyes); *β-cat4* (RNAi) h5w, 10.00±1.33 (SD; n = 10 eyes). *p<0.05, ***p<0.001 (t test). **(B)** Phototaxis assay of *β-cat4* (RNAi) intact animals. Graphical representation of the percentage of control and *β-cat4* (RNAi) planarians found in the different regions in 2 minutes. The scheme of the container with the different zones is shown. *β-cat4* (RNAi) animals became more insensitive to photophoby along the experiment. **(C)** FISH of *β-cat4* (red) in intact animals. Yellow arrows indicate isolated *β-cat4*+ cells in the trail posterior to the eyes, corresponding to eye precursor cells. **(D)** Quantification of *β-cat4+*, *sp6-9+* and *ovo+* cells in the eye of 7 days regenerating animals (n = 6 eyes). Scale bars = 50 μm.
(TIF)

S6 Fig *β-cat4* expression in eye progenitors and eye cells quantification during regeneration. FISH of *otxA* (red) and *sp6-9* (green) in control and *β-cat4* (RNAi) animals at indicated regeneration time points and its quantification in the eye structure. *otxA*+ cells in control R7d, 52.9±2.96 (SD; n = 10 eyes); *β-cat4* (RNAi) R7d, 21.00±1.58 (SD; n = 9 eyes); control R14d, 48.25±4.95 (SD; n = 8 eyes); *β-cat4* (RNAi) R14d, 15.80±0.92 (SD; n = 10 eyes). The *sp6-9*+ cells number for control R4d, 17.00±3.74 (SD; n = 6 eyes); *β-cat4* (RNAi) R4d, 18.75±3.01 (SD; n = 8 eyes); control R7d, 21.38±1.85 (SD; n = 8 eyes); *β-cat4* (RNAi) R7d, 17.00±2.78 (SD; n = 8 eyes); control R14d, 20.00±2.16 (SD; n = 10 eyes); *β-cat4* (RNAi) R14d, 9.20±2.20 (SD; n = 10 eyes). **p<0.01, ***p<0.001 (t test). Scale bar = 50 μm.
(TIF)

S7 Fig β-cat4 and β-cat1 are expressed in the nucleus of photoreceptor cells. **(A)** Left, scheme of a planarian eye showing the eye photoreceptors (in blue) and the pigment cells (in brown). middle, FISH of *β-cat1* (green) combined with immunostaining with anti-VC-1 (red), which labels rhabdomeres of photoreceptor cells (red), demonstrates the localization of *β-cat1* mRNA (green) in the photoreceptor area. Double FISH of *β-cat4* (red) and *β-cat1* (green) demonstrates their localization in the photoreceptor area (dashed line) **(B)** Immunostaining of control and *β-cat4* RNAi planarians with the anti- β-cat4 antibody (green) to demonstrate its specificity. Quantification of β-cat4 signal per cell shows that it decreases significantly in *β-cat4* RNAi animals compared to controls. ***p<0.001 (t test). n = 140 cells of 5 different animals per condition. β-cat4 signal was measured to obtain the raw integrated density (RID) for each individual nuclei. **(C)** Relative expression level of *β*-cat1 after *β-cat1 RNAi* by qRT-PCR. Values represent the means of three biological replicates. **(D)** FISH of *opsin* (red) and *tph* (green) in control and *β-cat1* (RNAi) at 9 days of regeneration. *β-cat1* (RNAi) planarians show larger eyes and the appearance of ectopic photoreceptor (yellow arrows) in 50% of the eyes analyzed. Nuclei are stained with DAPI. The respective quantification of *opsin*+ and *tph*+ cells per eye is shown. Ectopic cells were not included in the analysis. *opsin*+ cells in control R9d, 29±3 (SD; n = 8 eyes); *β-cat1* (RNAi) R9d, 38±8 (SD; n = 8 eyes). *tph*+ cells in control R9d, 13.5±3.5 (SD; n = 8 eyes); *β-cat1* (RNAi) R9d, 18,2±1.6 (SD; n = 8 eyes). *p<0.05, ***p<0.001 (t test). **(E)** Relative expression level of *β*-cat4 and APC after their *RNAi* by qRT-PCR. **(F)** Relative expression level of *β*-cat4 and *β*-cat1 after their *RNAi* by qRT-PCR. In **(D), (E)** and **(F)** values represent the means of three biological replicates. Error bars represent standard deviation. Data were analyzed by Student′s t-test. *p<0.05; **p<0.01. Scale bars = 20 μm (A, B), 50 μm (D).
(TIF)

S8 Fig *TCF-2* is involved in photoreceptor regeneration. **(A)** Phylogenetic analysis of TCF homologs from different species. Confidence values are shown at the main nodes. Accession number of the analyzed sequences: Aq-TCF ADO16566.1; NBf-TCF AAZ77711.1; Cs-TCF BAB68354.1; Dm-Pangolin P91943.1; Dr-LEF-1 NP_571501.1; Dr-TCF-1 NP_001012389.1; Dr-TCF-3 Q9YHE8.1; Dr-TCF-4 NP_571334.1; Eg-TCF-1b EUB55217.1; Eg-TCF-2 EUB60264.1; Eg-TCF3 CDS20600.1; Hs-LEF-1 NP_057353.1; Hs-TCF-1 AAH48769.1; Hs-TCF-3 NP_112573.1; Hs-TCF-4 NP_110383.2; Pd-TCF ANS60442.1; Sd-TCF CAH04889.1; Sp-Tcf/Lef AAD45010.1; Tc-Pangolin XP_008191151.1; Ts-TCF-1b OCK35857.1; Ts-TCF-2 OCK32932.1; Ts-TCF-3 OCK34187.1. TCF sequences from *planarian species* were found in Planmine [58]. Accession numbers of *Smed-TCF1*, *Smed-TCF2* and *Smed-TCF3* are: KY196226, KY196227, KY196228. Abbreviations: Aq, *Amphimedon queenslandica*; Bf, *Branchiostoma floridae*; Cs, *Ciona savignyi*; Dlac, *Dendrocelum lacteum*; Dm, Drosophila melanogaster; Dr, *Danio rerio*; Eg, Echinococcus granulosus; Hs, *Homo sapiens*; Pd, *Platynereis dumerilii*; Pnig, *Polycelis nigra*; Pten, *Polycelis tenuis*; Ptor, *Polycelis torva*; Sd, *Suberites domuncula*; Smed, *Schmidtea mediterranea*; Sp, *Strongylocentrotus purpuratus*; Spol, *Schmidtea polychroa*; Tc, *Tribolium castaneum*; Ts, *Taenia saginata*. **(B)** Expression pattern of *TCF-1* and *TCF-3*. Both of them are predominantly expressed in the CNS. **(C)** Relative expression level of *TCF-2* after *RNAi* by qRT-PCR. **(D)** Double FISH of *opsin* (red) and *tph* (green) in control and *TCF-2* (RNAi) animals at 5 days of regeneration with the corresponding quantification *opsin+* and *tph+* cells. *opsin*+ cells in control R5d, 25.38±2.07 (SD; n = 8 eyes); *TCF-2* (RNAi) R5d, 41.88±2.59 (SD; n = 8 eyes). *tph*+ cells number for control R5d, 14.38±1.77 (SD; n = 8 eyes); *TCF-2* (RNAi) R5d, 20.63±1.51 (SD; n = 8 eyes). ***p<0.001 (t test). **(E)** FISH of *pc2 (prohormone convertase 2)* (red) [103] in control and *TCF-2* (RNAi) at 7 days of regeneration. No difference is observed between control and *TCF-2* (RNAi) brains. **(F)** Relative expression levels of *TCF-2* and *β-cat4* after their *RNAi* by qRT-PCR. In **(C)** and **(F)** values represent the means of three biological replicates. Error bars represent standard deviation. Scale bar = 250 μm (B), 50 μm (C) and 200 μm (D).
(TIF)

S9 Fig Sequence alignments of TCFs proteins from different species. Alignment of the three *S*. *mediterranea* TCF proteins (Smed-TCF-1/3). The β-catenin binding domain [113], underlined in blue, is not conserved in TCF-1. The Groucho binding sequence [114], underlined in green, is not conserved in TCF-2. The HMG (High Mobility Group domain) [115], in red, and the NLS (Nuclear Localization Signal), are conserved in all TCFs. Accession numbers and abbreviations are found in S8 Fig legend.
(TIF)

S10 Fig Phylogenetic analysis of β-catenin proteins. β-catenin sequences from planarian species were identified in the Planmine database [58]. Platyhelminth species are squared in red. Planarian sequences are indicated with a planarian drawing. β-catenin proteins from species that show more than one β-catenin in the genome are labeled in yellow. From those, the ones that show a shorter C-terminal domain are marked in darker yellow. Accession number of the analyzed sequences: Ac-bcat NP_001191600.1; Aq-bcatA ADO16578.1; Aq-bcatB ADO16577.1; Bf-bcat XP_002588232.1; Ci-bcat BAA92185.1; Cl-bcat ABY21456.1; Crfor-bcat ADI48180.1; Cv-bcatAAL49497.1; Dm-Arm NP_476666.1; Dr-bcat1 NP_571134.2; Dr-bcat2 NP_001001889.1; Dr-Plak1 AAH58305.1, Dr-Plak2 XP_002665522.2; Hs-bcat NP_001895.1; Hs-Plak AAA64895.1; Hv-bcat AAQ02885.1; Mm-bcat NP_031640.1 NP_034723.1; Mm-Plak; Osc-bcat AEC12440.1; Pd-bcat ABQ85061.1; Ph-bcat; Sci-BcatB; Sci-BcatB; Sk-bcat NP_001158477.1; Sm-bcat1 XP_018651394.1; Sm-bcat2 XP_018646608.1, Sm-bcat3/4 XP_018649674.1; Smed-bcat1 ABW79875.1; Smed-bcat2 ABW79874.1; Sp-bcat NP_001027543.1; Tc-Arm1 EFA10737.1; Tc-Arm2 NP_001164124.1. β-Catenin sequences from *Echinococcus multilocularis* and *Kronborgia cf*. *amphipodicola* were found in the available databases [95, 96]. β-catenin sequences from planarian species were found in the Planmine [58]. Accession numbers of the new planarian β-catenins are: β-cat3, KY196224; and β-cat4, KY196225. Abbreviations: Ac, *Aplysia californica*; Aq, *Amphimedon queenslandica*; Bf, *Branchiostoma floridae*; Ci, *Ciona intestinalis*; Cl, *Cerebratulus lacteus*; Crfor, *Crepidula fornicata*; Cv, *Chaetopterus variopedatus*; Dm, *Drosophila melanogaster*; Dr, *Danio rerio*; Em, *Echinococcus multilocularis*; Hs, *Homo sapiens*; Hv, *Hydra vulgaris*; Kram, *Kronborgia amphipodicola*; Mm, *Mus musculus*; Osc, *Oscarella carmela*; Pd, *Platynereis dumerilii*; Ph, *Parhyale hawaiensis*; Pnig, *Polycelis nigra*; Pten, *Polycelis tenuis*; Sci, *Sycon ciliatum*; Sk, *Saccoglossus kowalevskii*; Sm, *Schistosoma mansoni*; Smed, *Schmidtea mediterranea*; Sp, *Strongylocentrotus purpuratus*; Spol, *Schmidtea polychroa*; Tc, *Tribolium castaneum*.
(TIF)

S11 Fig Alignment of β-catenin protein sequences which show a shorter C-terminal transactivation domain. The functional domains are indicated. Arm splicing isoform (Neural Arm, accession number AAB58731.1) and Plakoglobin, highlighted in yellow, conserve all functional domains but show a shorter C-terminal transactivation domain. Accession numbers and abbreviations are indicated in S10 Fig.
(PDF)

S1 Movie Movie showing the proper negative phototaxis behavior of control animals when exposed to light. Control animals moved away from the light and remained in the darkest zone (zone 3 in Fig 2).
(AVI)

S2 Movie Movie showing that *β-cat4* (RNAi) animals do not show the proper negative phototaxis behavior when exposed to light. Although *β-cat4* (RNAi) organisms seemed to move normally, most of them remained in the clearest zone and did not reach the darkest zone in the same time period than controls (S1 Movie).
(AVI)

S3 Movie Movie showing the animated Z-Stack of control animals stained with *opsin* and *tph*. Animated Z-Stack of the eye corresponding to a control animal. Nuclei are labeled with DAPI (blue), photoreceptors are labeled with an RNA probe of *opsin* (red) and pigment cells are labeled with an RNA probe of *tph* (green). Scale bars = 10 μm.
(AVI)

S4 Movie Movie showing the animated Z-Stack of a *β-cat1* (RNAi) animal stained with *opsin* and *tph*. Animated Z-Stack of the eye corresponding to a *β-cat1* (RNAi) animal. Nuclei are labeled with DAPI (blue), photoreceptors are labeled with an RNA probe of *opsin* (red) and pigment cells are labeled with an RNA probe of *tph* (green). Scale bars = 10 μm.
(AVI)

S1 Table Primers used for dsRNA and ssRNA synthesis and for qPCR analysis. (PDF)
