# Supplementary figures and images for: A C-terminally truncated form of β-catenin acts as a novel regulator of Wnt/β-catenin signaling in planarians
Source: PLoS Genet. 2017 Oct 4;13(10):e1007030. doi: 10.1371/journal.pgen.1007030 (PMC5643146; doi:10.1371/journal.pgen.1007030)

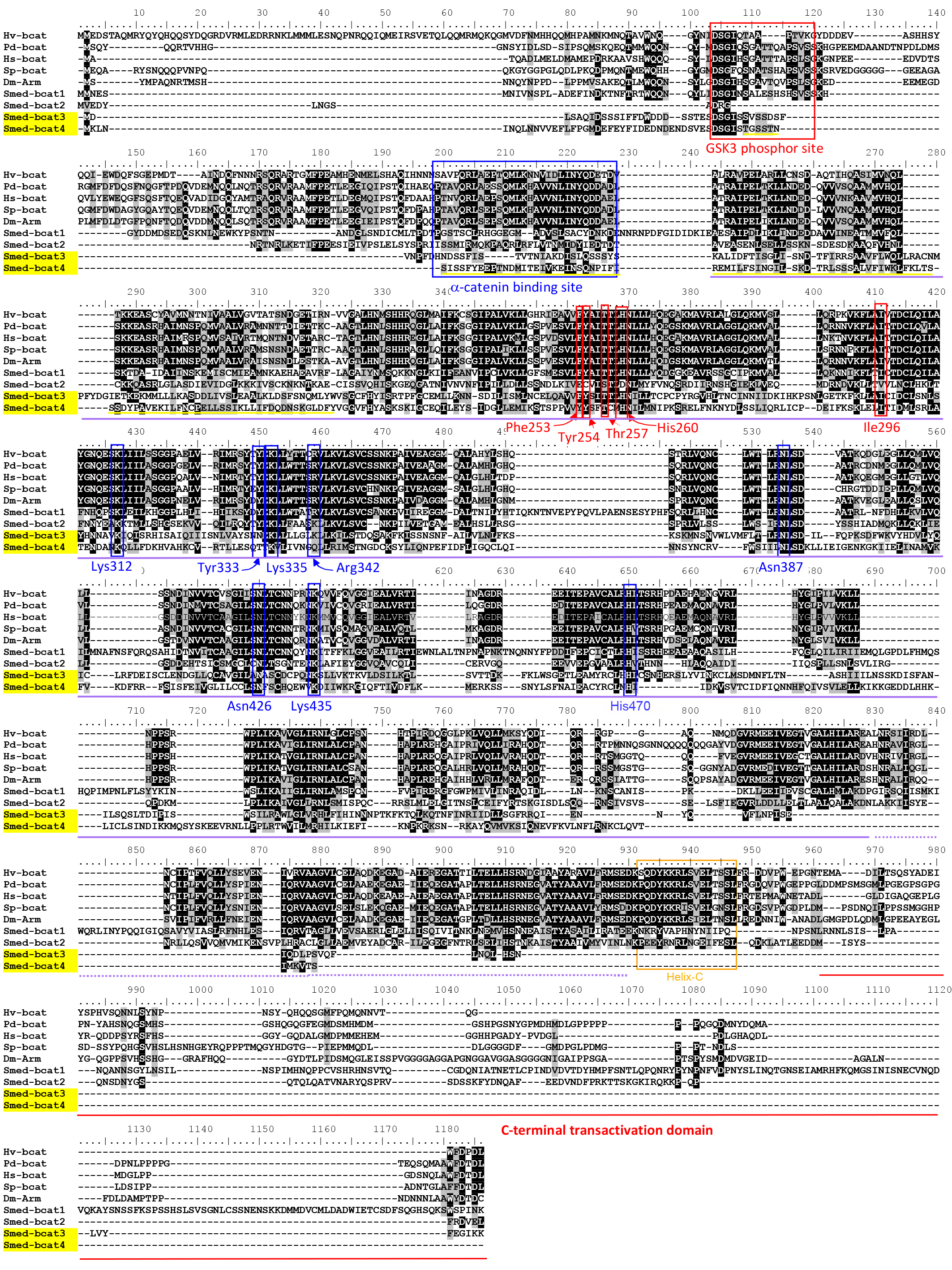

Supplement: S1 Fig — Protein regions and amino acids required for Wnt signaling are indicated in red. β-cat3 and 4 conserve the GSK3 phosphorylation sites in the N terminus, the hydrophobic pocket formed by Phe 253 and some of the surrounding residues, essential for Axin and TCF binding [105]. The red line underlies the C-terminal transactivation domain, which is necessary and sufficient for signaling through TCF factors [106, 107]. The C-terminal transactivation domain is lost in β-cat3 and 4 proteins. Amino acids required for cell-cell adhesion but also involved in TCF interaction, which are conserved in β-cat3 and 4 proteins, are labeled in blue: the armadillo repeats 4–9 in human β-catenin constitute a core interacting platform for cadherin and TCF binding, in which Lys312 and Lys435 form the critical salt bridges [108, 109]. The α-catenin binding sites [110, 111] appear conserved in β-cat4 but not in β-cat3. Armadillo repeats are underlined with a violet line. The conserved two last repeats, which are not present in β-cat3 and 4 proteins, are underlined by a dashed line. The Helix-C (orange square), a helix-α structure found C-terminal to the last Armadillo repeat and required for transcriptional co-activation [112], is also not conserved in β-cat3 and 4 proteins. The amino acids used to generate the anti β-cat4 antibody are underlined in yellow. Accession numbers of the sequences analyzed are: Hv-bcat, AAQ02885.1; Pd-bcat, ABQ85061.1; Hs-bcat, NP_001895.1; Sp-bcat, NP_001027543.1; Dm-Arm, NP_476666.1; Smed-bcat1, ABW79875.1; Smed-bcat2, ABW79874.1. The accession numbers of the new planarian β-catenins are: β-cat3, KY196224; and β-cat4, KY196225. Abbreviations: Hv, Hidra vulgaris; Pd, P. dumerilii; Hs, H. spiens; Sp, Strongilocentrotus purpuratus; Dm, Drosophila melanogaster; Smed, S. mediterranea. (TIF) [file pgen.1007030.s001.tif]

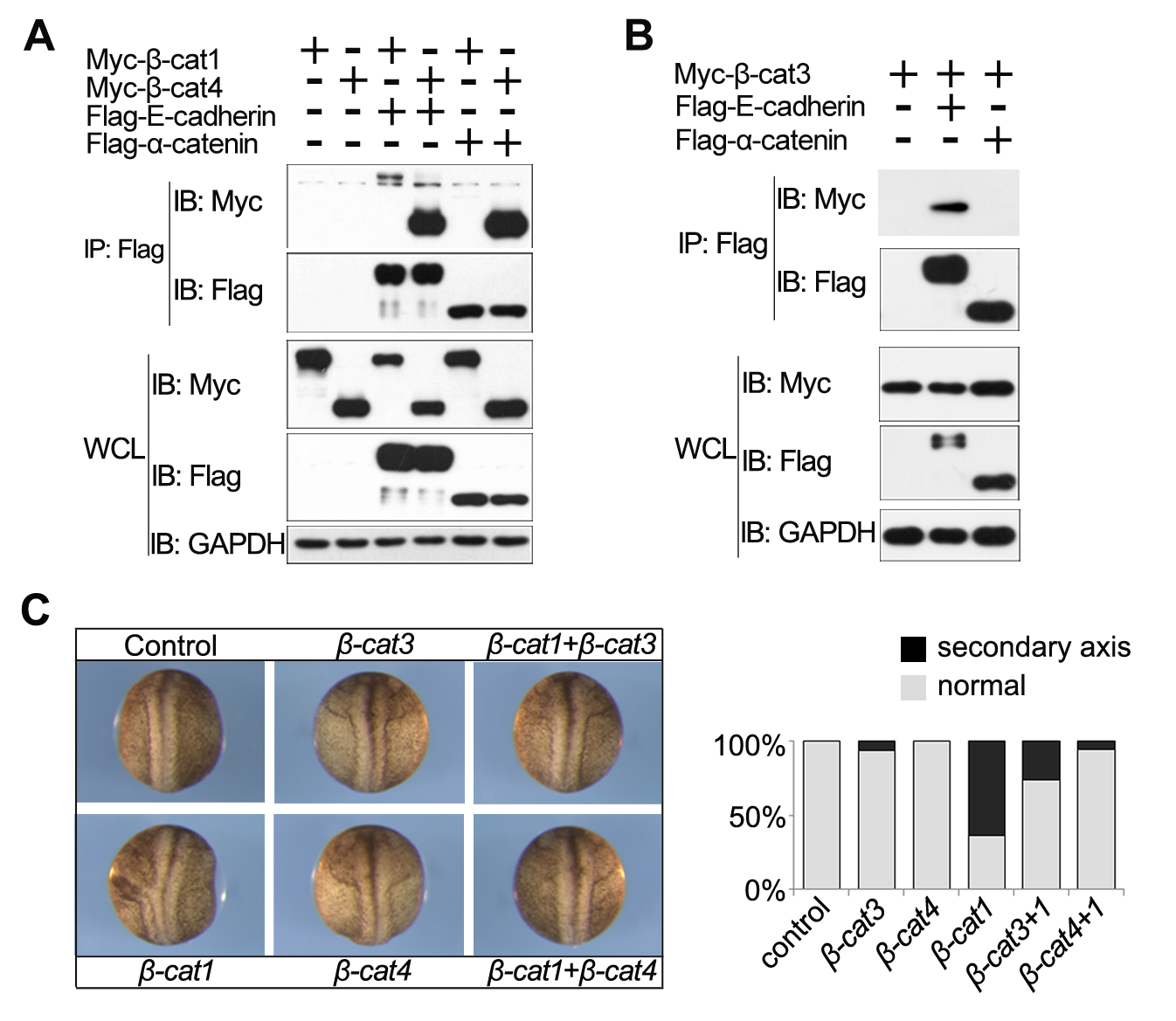

Supplement: S2 Fig — (A-B) Co-immunoprecipitation assays in 293T cells showed the ability of β-cat4 to interact with E-cadherin and α-catenin, while β-cat3 could only interact with E-cadherin. β-cat1 was used as a control since it is reported its interaction with E-cadherin [40]. (C) Axis duplication assays in Xenopus embryos. β-cat1 mRNA injection induces a secondary axis in Xenopus embryos, as reported [32, 40], whereas no effects are observed after β-cat3 or 4 mRNA injection. However co-injection of β-cat3/4 together with β-cat1 rescued the “double axis” phenotype. The percentages of embryos with the indicated phenotypes are shown (n = 50). (TIF) [file pgen.1007030.s002.tif]

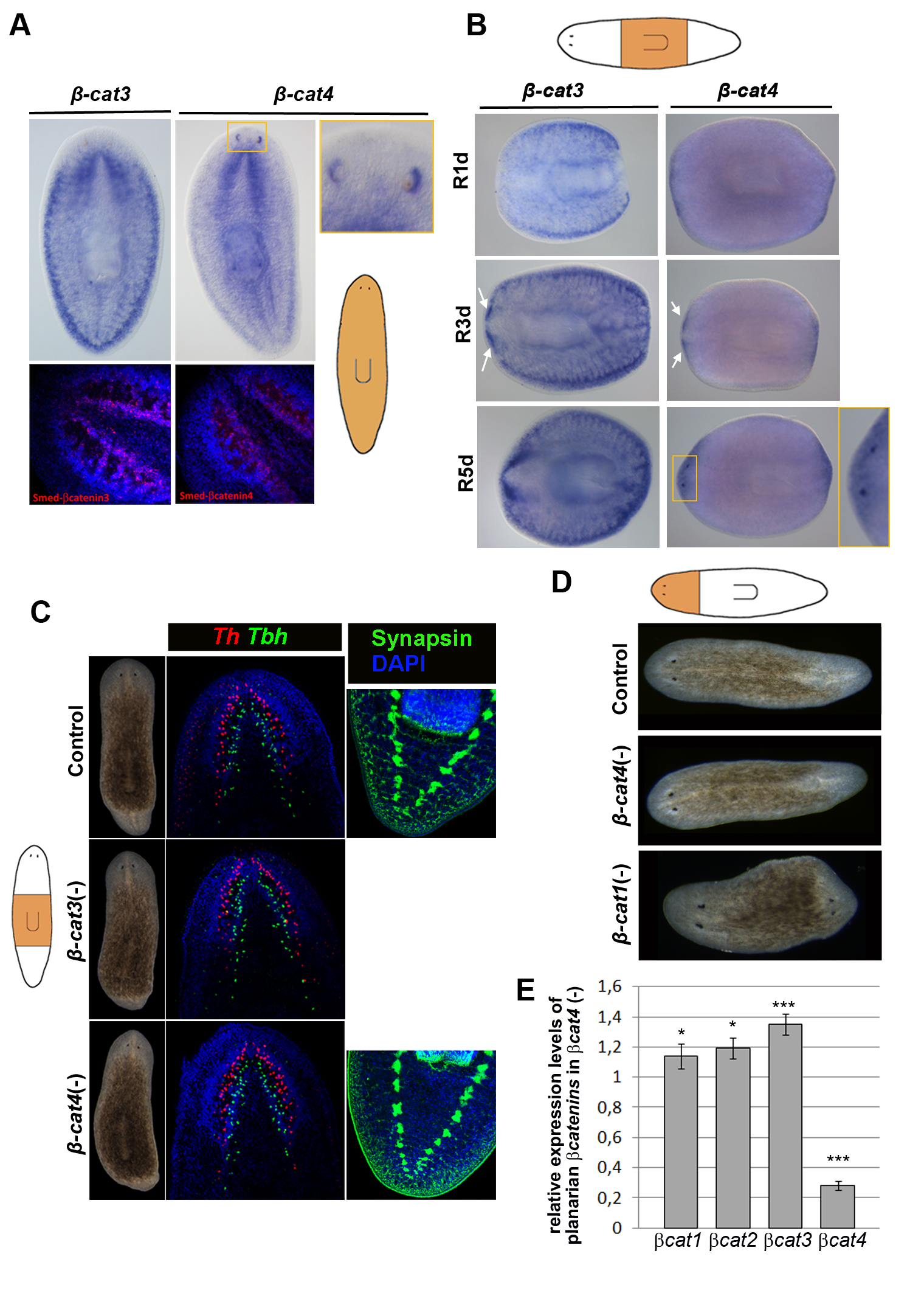

Supplement: S3 Fig — (A) Both β-cat3 and β-cat4 were mainly expressed in the CNS after WISH and FISH (red). β-cat4 was also expressed in photoreceptors (see magnification in the orange box). Analyzed planarians correspond to intact animals. (B) β-cat3 and β-cat4 expression pattern during regeneration of trunk fragments, which must regenerate the head and the tail. β-cat3 and β-cat4 were expressed in the newly formed brain (arrows in R3d) and β-cat4 is also expressed in the regenerating eyes (see magnification in the orange box). (C) Phenotype of control and β-cat3 and 4 RNAi animals after dsRNA injection and induced regeneration. No polarity defects were observed in β-cat3 or β-cat4 RNAi animals. No obvious affection in the brain was neither observed through analysis of brain markers, 3C11 (green), Smed-th (tyrosine hydroxylase) (red), and Smed-tbh (tryptophan hydroxylase) (green) [102]. Planarians shown were trunk fragments at 12 days of regeneration. (D) β-cat1 RNAi caused anteriorization of regenerating head fragments, as expected [31–33], while β-cat4 RNAi planarians show a normal regenerated posterior region, indicating that β-cat4 RNAi does not cause antero-posterior defects. (E) Relative expression levels of β-cat1/2/3/4 after β-cat4 RNAi by qRT-PCR. Values represent the means of three biological replicates. Error bars represent standard deviation. Data were analyzed by Student′s t-test. *p<0.05; **p<0.01; ***p<0.001. Scale bars = 250 μm (A, B, C, D) and 50 μm (magnification in A and B). (TIF) [file pgen.1007030.s003.tif]

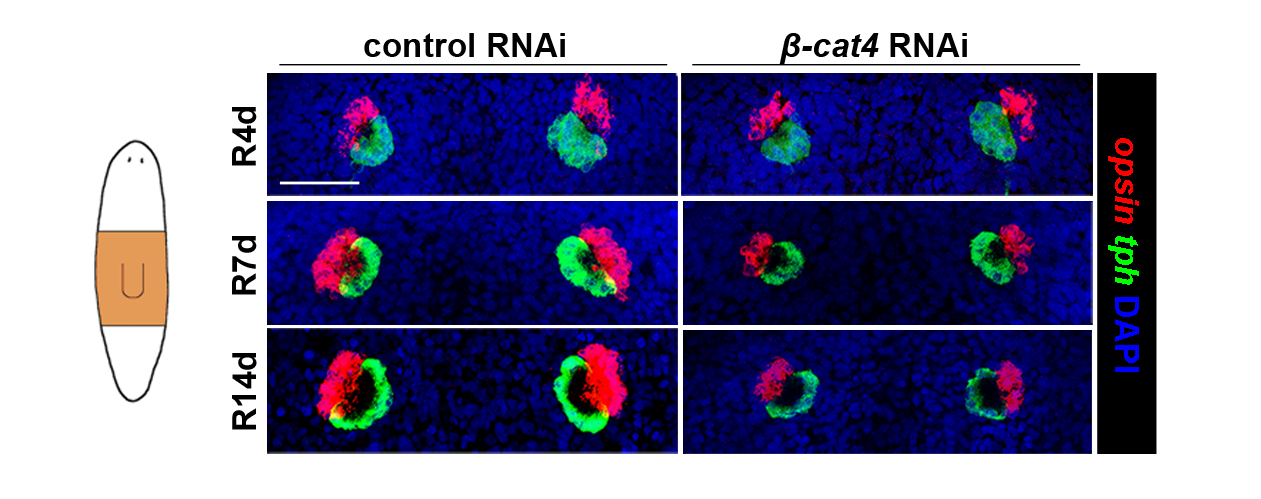

Supplement: S4 Fig — Double FISH of opsin (red) and tph (green) in control and β-cat4 (RNAi) animals, at 4, 7, and 14 days of regeneration. Scale bar = 50 μm. (TIF) [file pgen.1007030.s004.tif]

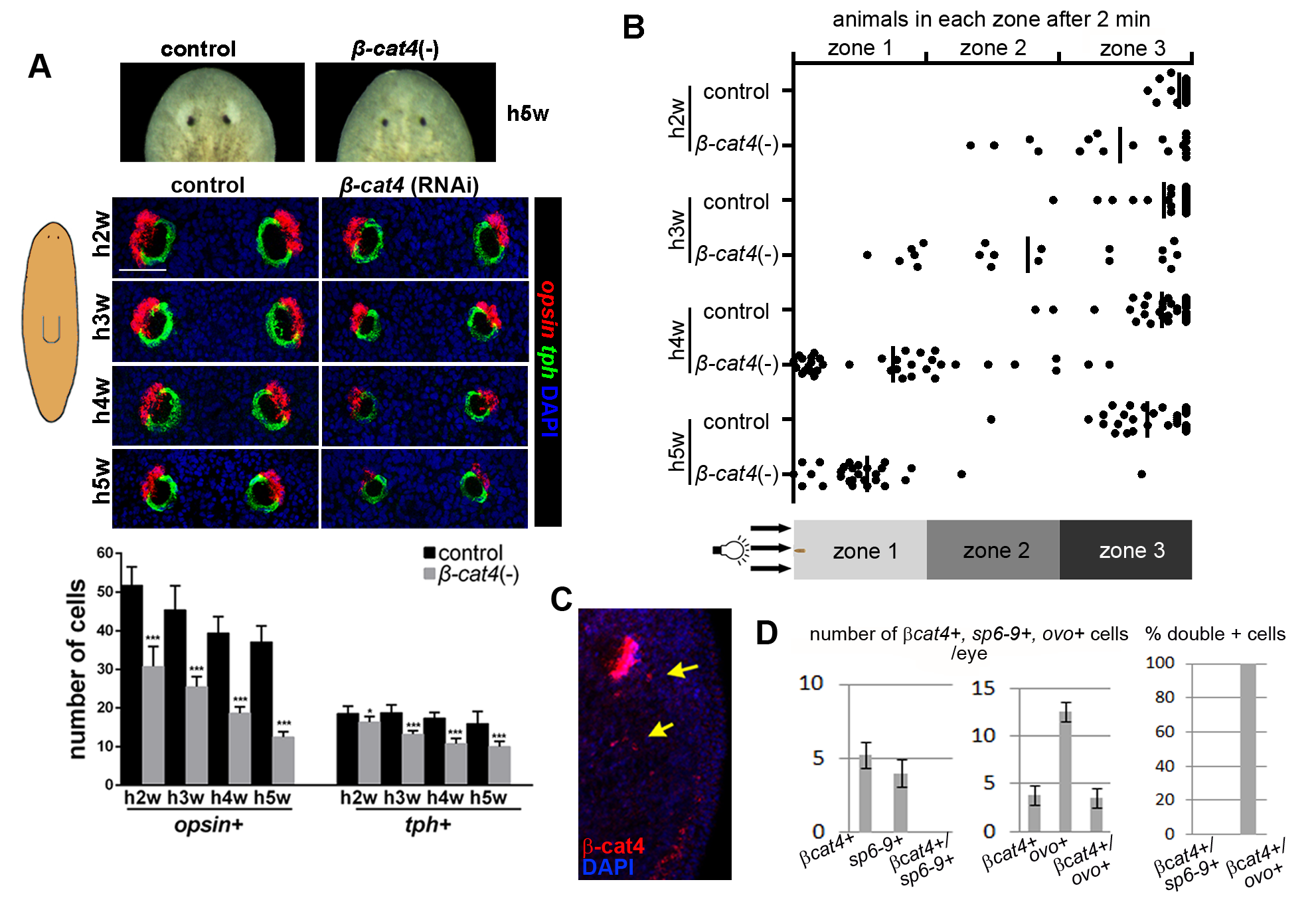

Supplement: S5 Fig — (A) β-cat4 (RNAi) planarians after 5 weeks injection show smaller eyes with small photoreceptor area. FISH of opsin (red) and tph (green) in control and β-cat4 (RNAi) intact animals along the 5 weeks of β-cat4 dsRNA injection and its quantification, showing a decrease mainly in photoreceptor cells. opsin+ cells in control h2w, 51.80±4.71 (SD; n = 10 eyes); β-cat4 (RNAi) h2w, 30.75±5.20 (SD; n = 8 eyes); control h3w, 45.40±6.26 (SD; n = 10 eyes); β-cat4 (RNAi) h3w, 25.50±2.62 (SD; n = 8 eyes); control h4w, 39.40±4.25 (SD; n = 10 eyes); β-cat4 (RNAi) h4w, 18.70±1.64 (SD; n = 10 eyes); control h5w, 37.08±4.14 (SD; n = 10 eyes); β-cat4 (RNAi) h5w, 12.50±1.35 (SD; n = 10 eyes). tph+ cells in control h2w, 18.60±1.90 (SD; n = 10 eyes); β-cat4 (RNAi) h2w, 16.38±1.41 (SD; n = 8 eyes); control h3w, 18.80±2.00 (SD; n = 10 eyes); β-cat4 (RNAi) h3w, 13.25±0.89 (SD; n = 8 eyes); control h4w, 17.40±1.43 (SD; n = 10 eyes); β-cat4 (RNAi) h4w, 10.80±1.32 (SD; n = 10 eyes); control h5w, 16.00±3.10 (SD; n = 12 eyes); β-cat4 (RNAi) h5w, 10.00±1.33 (SD; n = 10 eyes). *p<0.05, ***p<0.001 (t test). (B) Phototaxis assay of β-cat4 (RNAi) intact animals. Graphical representation of the percentage of control and β-cat4 (RNAi) planarians found in the different regions in 2 minutes. The scheme of the container with the different zones is shown. β-cat4 (RNAi) animals became more insensitive to photophoby along the experiment. (C) FISH of β-cat4 (red) in intact animals. Yellow arrows indicate isolated β-cat4+ cells in the trail posterior to the eyes, corresponding to eye precursor cells. (D) Quantification of β-cat4+, sp6-9+ and ovo+ cells in the eye of 7 days regenerating animals (n = 6 eyes). Scale bars = 50 μm. (TIF) [file pgen.1007030.s005.tif]

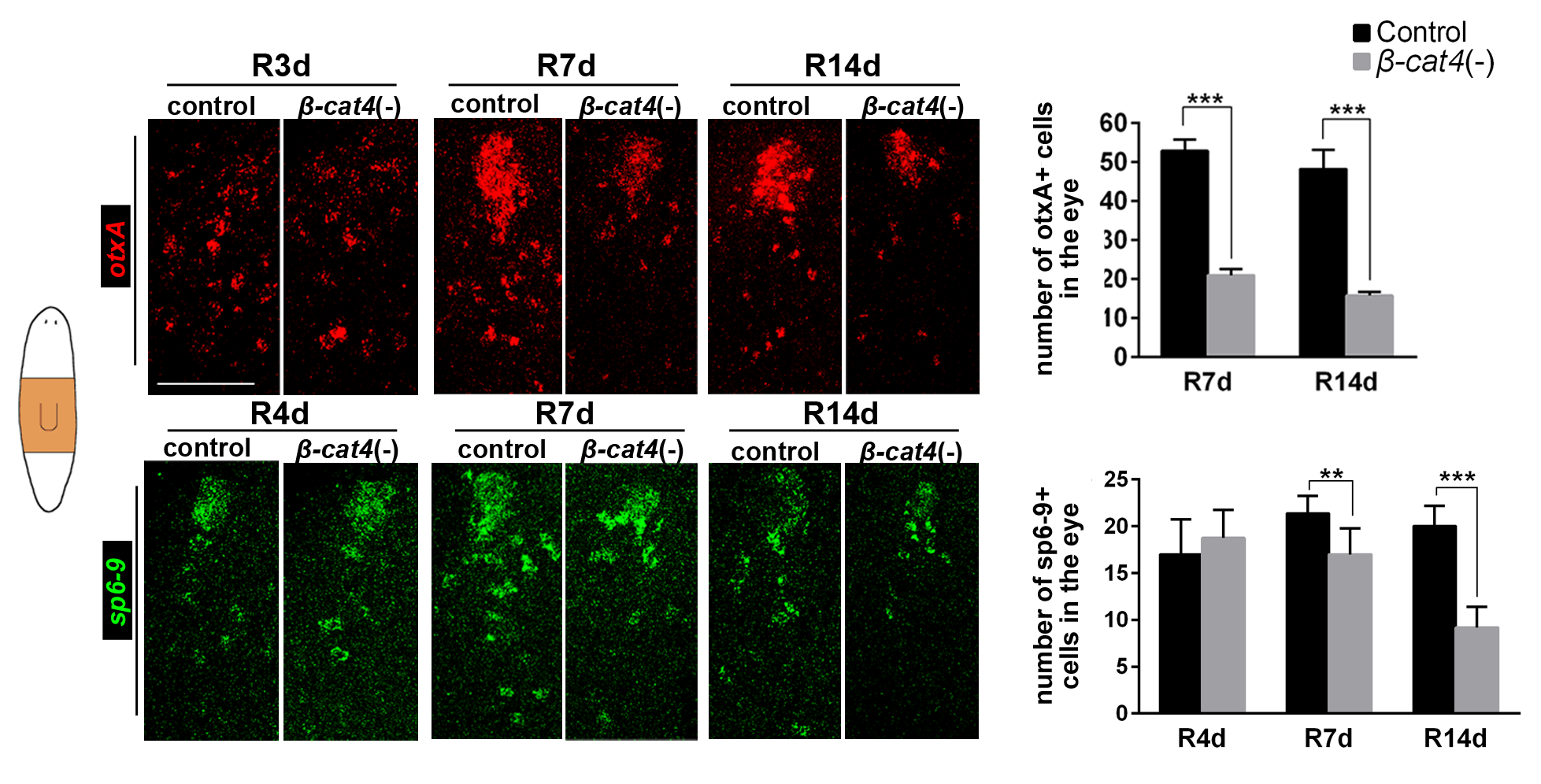

Supplement: S6 Fig — FISH of otxA (red) and sp6-9 (green) in control and β-cat4 (RNAi) animals at indicated regeneration time points and its quantification in the eye structure. otxA+ cells in control R7d, 52.9±2.96 (SD; n = 10 eyes); β-cat4 (RNAi) R7d, 21.00±1.58 (SD; n = 9 eyes); control R14d, 48.25±4.95 (SD; n = 8 eyes); β-cat4 (RNAi) R14d, 15.80±0.92 (SD; n = 10 eyes). The sp6-9+ cells number for control R4d, 17.00±3.74 (SD; n = 6 eyes); β-cat4 (RNAi) R4d, 18.75±3.01 (SD; n = 8 eyes); control R7d, 21.38±1.85 (SD; n = 8 eyes); β-cat4 (RNAi) R7d, 17.00±2.78 (SD; n = 8 eyes); control R14d, 20.00±2.16 (SD; n = 10 eyes); β-cat4 (RNAi) R14d, 9.20±2.20 (SD; n = 10 eyes). **p<0.01, ***p<0.001 (t test). Scale bar = 50 μm. (TIF) [file pgen.1007030.s006.tif]

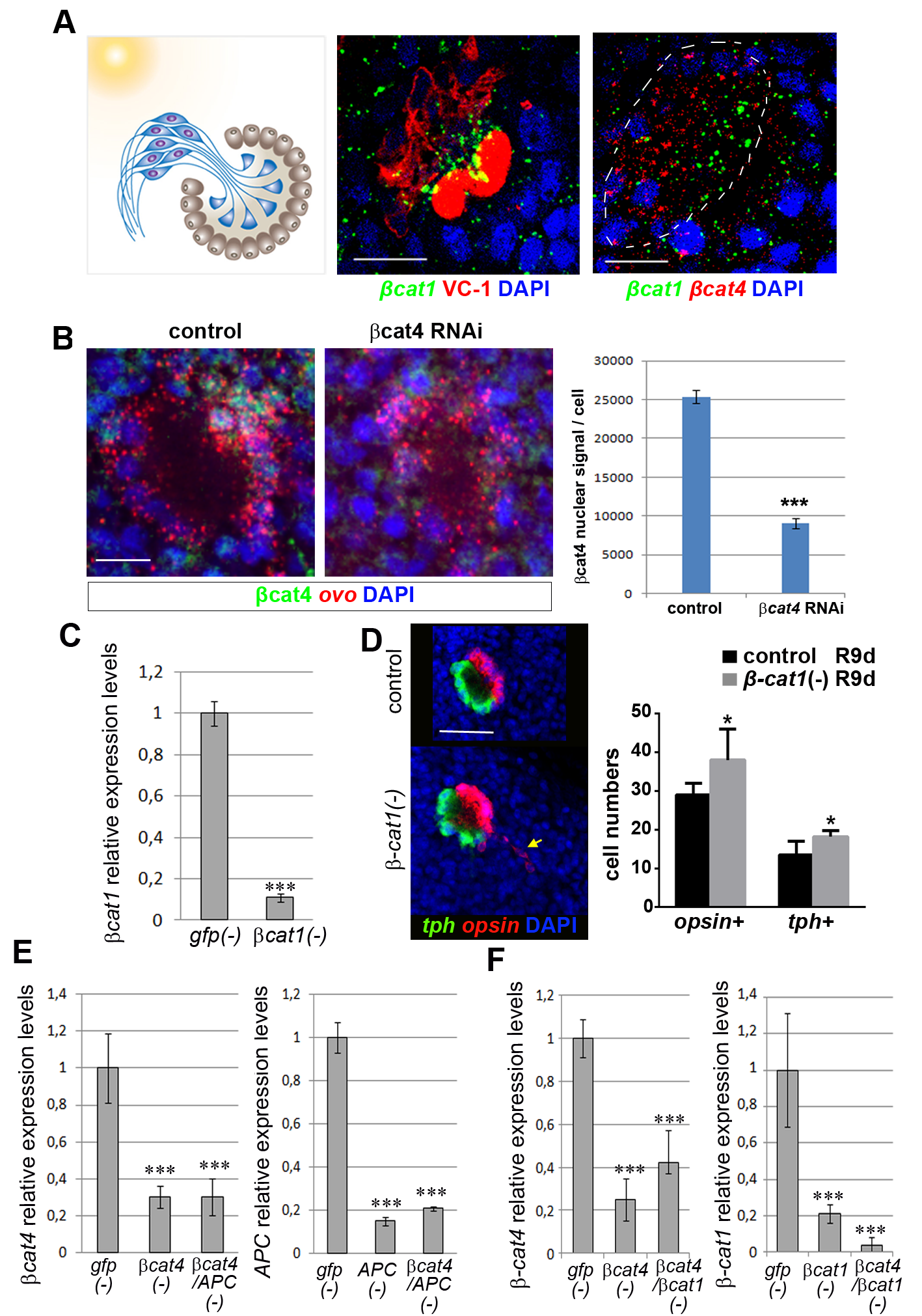

Supplement: S7 Fig — (A) Left, scheme of a planarian eye showing the eye photoreceptors (in blue) and the pigment cells (in brown). middle, FISH of β-cat1 (green) combined with immunostaining with anti-VC-1 (red), which labels rhabdomeres of photoreceptor cells (red), demonstrates the localization of β-cat1 mRNA (green) in the photoreceptor area. Double FISH of β-cat4 (red) and β-cat1 (green) demonstrates their localization in the photoreceptor area (dashed line) (B) Immunostaining of control and β-cat4 RNAi planarians with the anti- β-cat4 antibody (green) to demonstrate its specificity. Quantification of β-cat4 signal per cell shows that it decreases significantly in β-cat4 RNAi animals compared to controls. ***p<0.001 (t test). n = 140 cells of 5 different animals per condition. β-cat4 signal was measured to obtain the raw integrated density (RID) for each individual nuclei. (C) Relative expression level of β-cat1 after β-cat1 RNAi by qRT-PCR. Values represent the means of three biological replicates. (D) FISH of opsin (red) and tph (green) in control and β-cat1 (RNAi) at 9 days of regeneration. β-cat1 (RNAi) planarians show larger eyes and the appearance of ectopic photoreceptor (yellow arrows) in 50% of the eyes analyzed. Nuclei are stained with DAPI. The respective quantification of opsin+ and tph+ cells per eye is shown. Ectopic cells were not included in the analysis. opsin+ cells in control R9d, 29±3 (SD; n = 8 eyes); β-cat1 (RNAi) R9d, 38±8 (SD; n = 8 eyes). tph+ cells in control R9d, 13.5±3.5 (SD; n = 8 eyes); β-cat1 (RNAi) R9d, 18,2±1.6 (SD; n = 8 eyes). *p<0.05, ***p<0.001 (t test). (E) Relative expression level of β-cat4 and APC after their RNAi by qRT-PCR. (F) Relative expression level of β-cat4 and β-cat1 after their RNAi by qRT-PCR. In (D), (E) and (F) values represent the means of three biological replicates. Error bars represent standard deviation. Data were analyzed by Student′s t-test. *p<0.05; **p<0.01. Scale bars = 20 μm (A, B), 50 μm (D). (TIF) [file pgen.1007030.s007.tif]

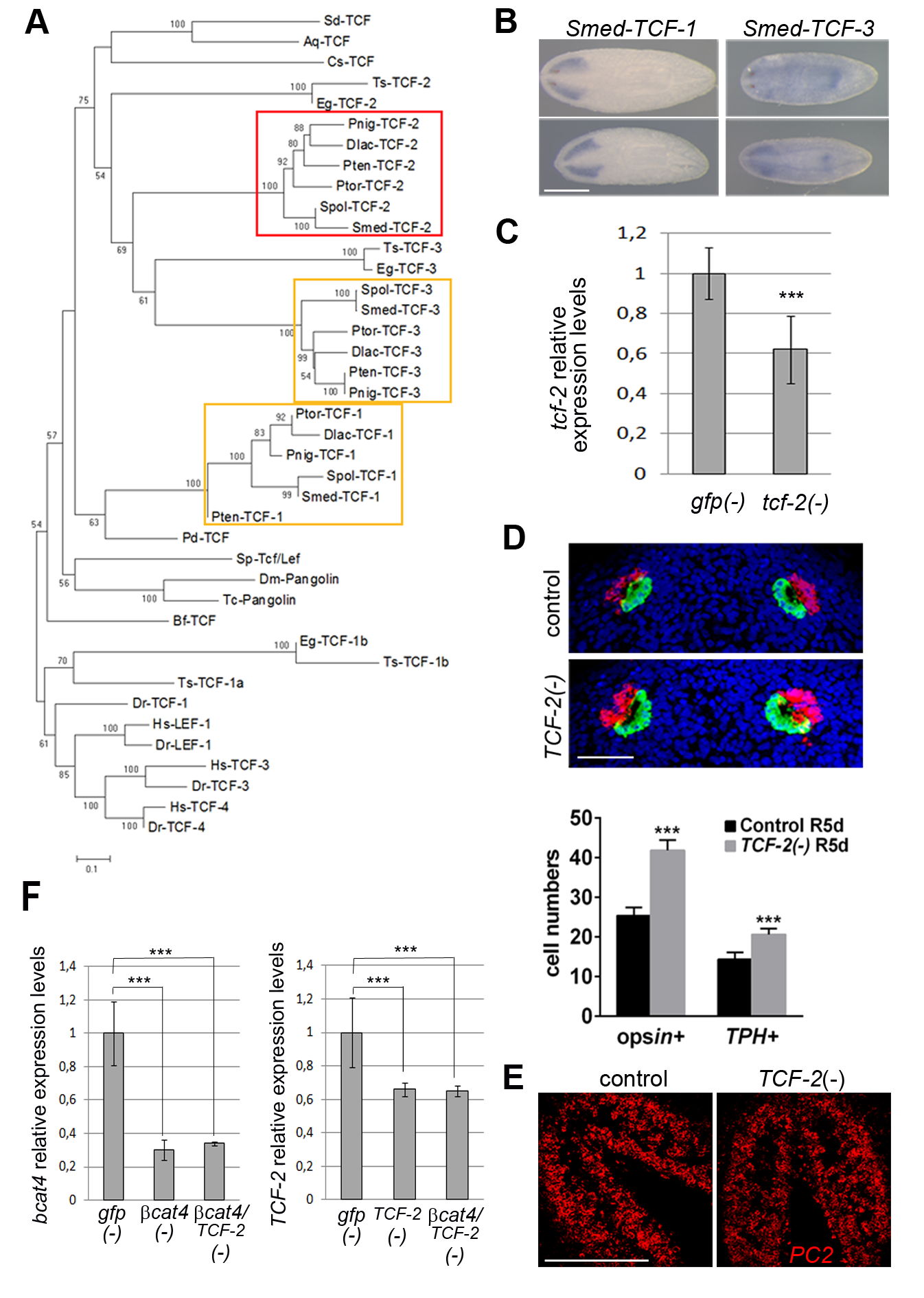

Supplement: S8 Fig — (A) Phylogenetic analysis of TCF homologs from different species. Confidence values are shown at the main nodes. Accession number of the analyzed sequences: Aq-TCF ADO16566.1; NBf-TCF AAZ77711.1; Cs-TCF BAB68354.1; Dm-Pangolin P91943.1; Dr-LEF-1 NP_571501.1; Dr-TCF-1 NP_001012389.1; Dr-TCF-3 Q9YHE8.1; Dr-TCF-4 NP_571334.1; Eg-TCF-1b EUB55217.1; Eg-TCF-2 EUB60264.1; Eg-TCF3 CDS20600.1; Hs-LEF-1 NP_057353.1; Hs-TCF-1 AAH48769.1; Hs-TCF-3 NP_112573.1; Hs-TCF-4 NP_110383.2; Pd-TCF ANS60442.1; Sd-TCF CAH04889.1; Sp-Tcf/Lef AAD45010.1; Tc-Pangolin XP_008191151.1; Ts-TCF-1b OCK35857.1; Ts-TCF-2 OCK32932.1; Ts-TCF-3 OCK34187.1. TCF sequences from planarian species were found in Planmine [58]. Accession numbers of Smed-TCF1, Smed-TCF2 and Smed-TCF3 are: KY196226, KY196227, KY196228. Abbreviations: Aq, Amphimedon queenslandica; Bf, Branchiostoma floridae; Cs, Ciona savignyi; Dlac, Dendrocelum lacteum; Dm, Drosophila melanogaster; Dr, Danio rerio; Eg, Echinococcus granulosus; Hs, Homo sapiens; Pd, Platynereis dumerilii; Pnig, Polycelis nigra; Pten, Polycelis tenuis; Ptor, Polycelis torva; Sd, Suberites domuncula; Smed, Schmidtea mediterranea; Sp, Strongylocentrotus purpuratus; Spol, Schmidtea polychroa; Tc, Tribolium castaneum; Ts, Taenia saginata. (B) Expression pattern of TCF-1 and TCF-3. Both of them are predominantly expressed in the CNS. (C) Relative expression level of TCF-2 after RNAi by qRT-PCR. (D) Double FISH of opsin (red) and tph (green) in control and TCF-2 (RNAi) animals at 5 days of regeneration with the corresponding quantification opsin+ and tph+ cells. opsin+ cells in control R5d, 25.38±2.07 (SD; n = 8 eyes); TCF-2 (RNAi) R5d, 41.88±2.59 (SD; n = 8 eyes). tph+ cells number for control R5d, 14.38±1.77 (SD; n = 8 eyes); TCF-2 (RNAi) R5d, 20.63±1.51 (SD; n = 8 eyes). ***p<0.001 (t test). (E) FISH of pc2 (prohormone convertase 2) (red) [103] in control and TCF-2 (RNAi) at 7 days of regeneration. No difference is observed between control and TCF-2 (RNAi) brains. ( [file pgen.1007030.s008.tif]

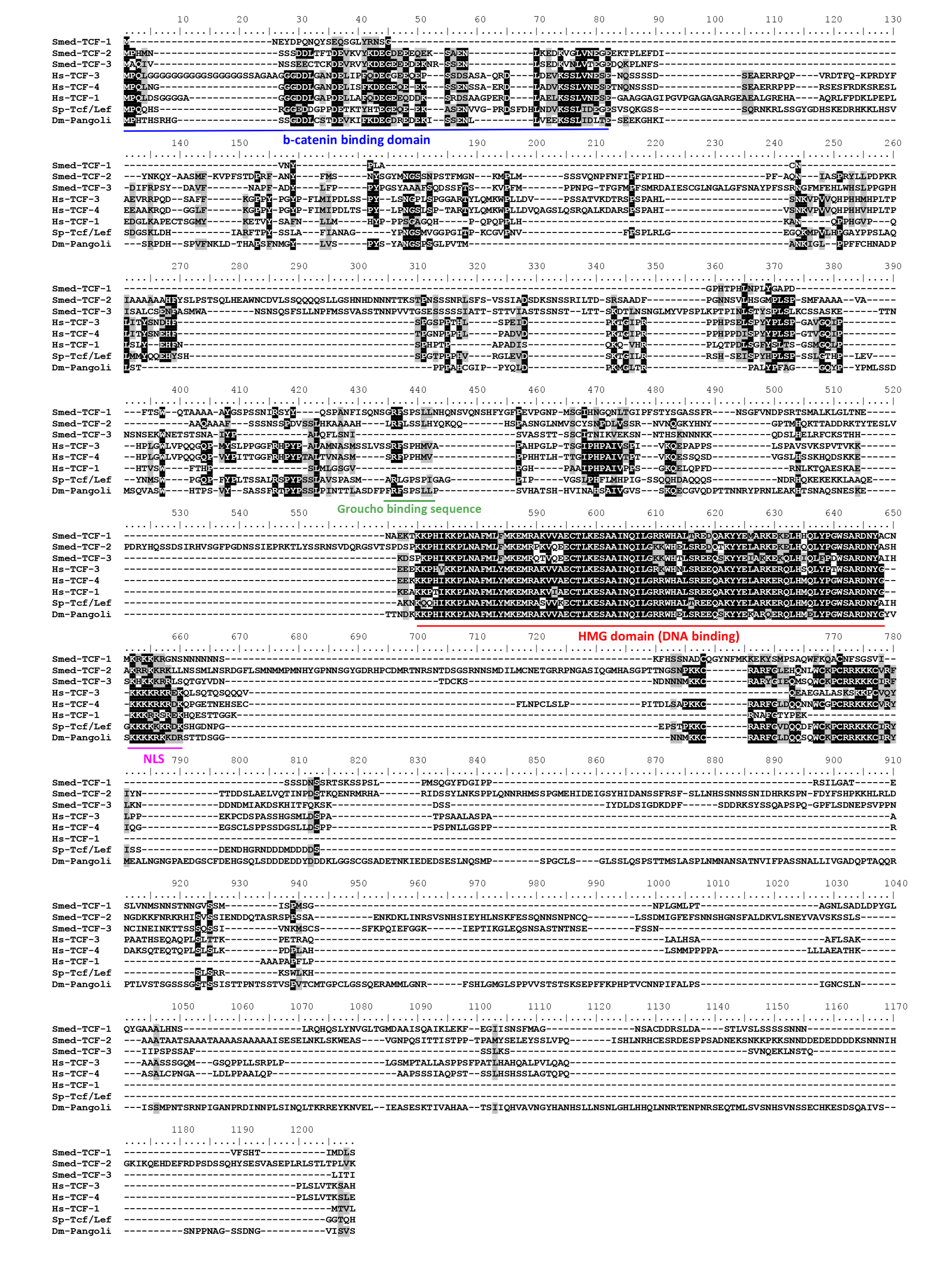

Supplement: S9 Fig — Alignment of the three S. mediterranea TCF proteins (Smed-TCF-1/3). The β-catenin binding domain [113], underlined in blue, is not conserved in TCF-1. The Groucho binding sequence [114], underlined in green, is not conserved in TCF-2. The HMG (High Mobility Group domain) [115], in red, and the NLS (Nuclear Localization Signal), are conserved in all TCFs. Accession numbers and abbreviations are found in S8 Fig legend. (TIF) [file pgen.1007030.s009.tif]

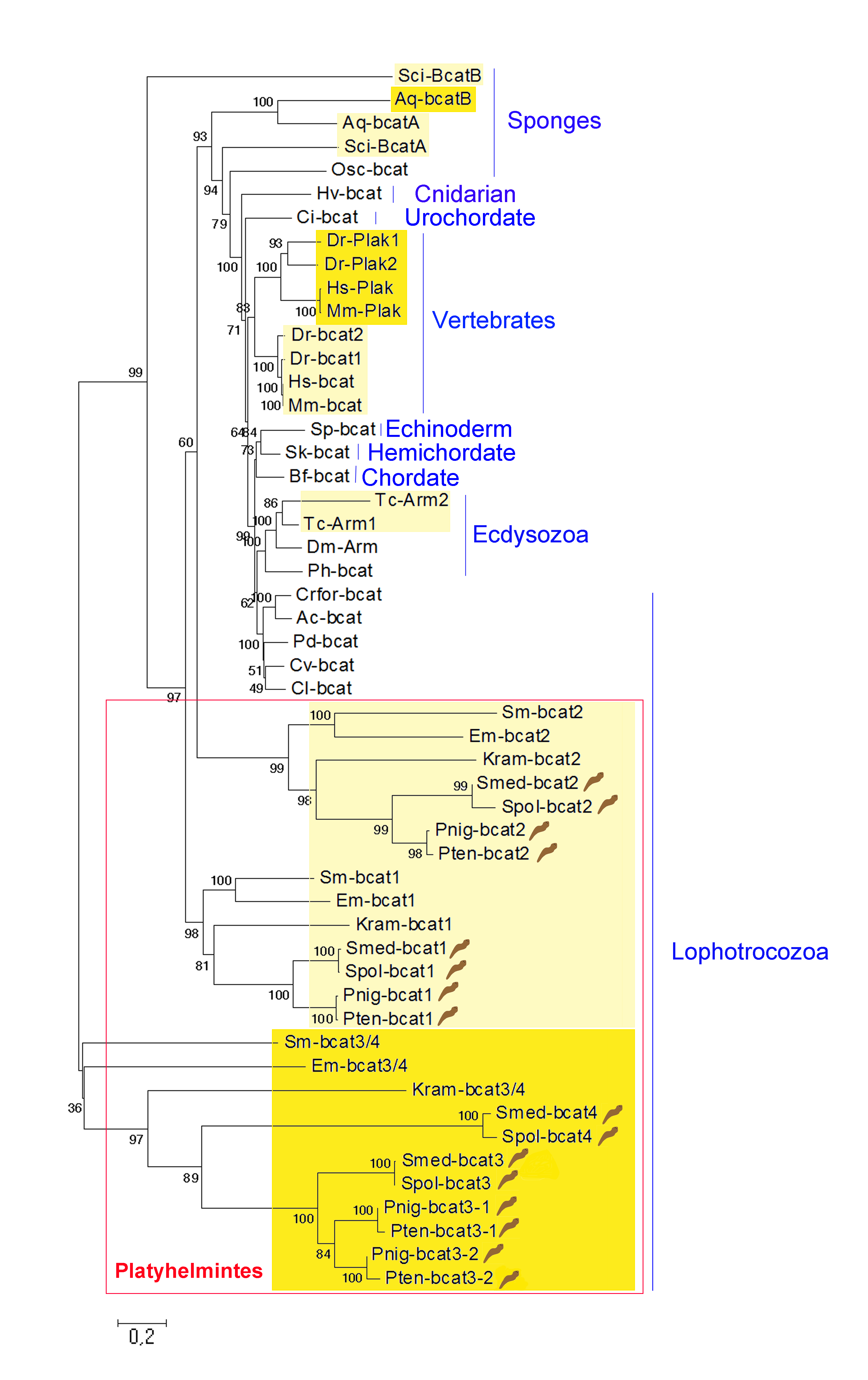

Supplement: S10 Fig — β-catenin sequences from planarian species were identified in the Planmine database [58]. Platyhelminth species are squared in red. Planarian sequences are indicated with a planarian drawing. β-catenin proteins from species that show more than one β-catenin in the genome are labeled in yellow. From those, the ones that show a shorter C-terminal domain are marked in darker yellow. Accession number of the analyzed sequences: Ac-bcat NP_001191600.1; Aq-bcatA ADO16578.1; Aq-bcatB ADO16577.1; Bf-bcat XP_002588232.1; Ci-bcat BAA92185.1; Cl-bcat ABY21456.1; Crfor-bcat ADI48180.1; Cv-bcatAAL49497.1; Dm-Arm NP_476666.1; Dr-bcat1 NP_571134.2; Dr-bcat2 NP_001001889.1; Dr-Plak1 AAH58305.1, Dr-Plak2 XP_002665522.2; Hs-bcat NP_001895.1; Hs-Plak AAA64895.1; Hv-bcat AAQ02885.1; Mm-bcat NP_031640.1 NP_034723.1; Mm-Plak; Osc-bcat AEC12440.1; Pd-bcat ABQ85061.1; Ph-bcat; Sci-BcatB; Sci-BcatB; Sk-bcat NP_001158477.1; Sm-bcat1 XP_018651394.1; Sm-bcat2 XP_018646608.1, Sm-bcat3/4 XP_018649674.1; Smed-bcat1 ABW79875.1; Smed-bcat2 ABW79874.1; Sp-bcat NP_001027543.1; Tc-Arm1 EFA10737.1; Tc-Arm2 NP_001164124.1. β-Catenin sequences from Echinococcus multilocularis and Kronborgia cf. amphipodicola were found in the available databases [95, 96]. β-catenin sequences from planarian species were found in the Planmine [58]. Accession numbers of the new planarian β-catenins are: β-cat3, KY196224; and β-cat4, KY196225. Abbreviations: Ac, Aplysia californica; Aq, Amphimedon queenslandica; Bf, Branchiostoma floridae; Ci, Ciona intestinalis; Cl, Cerebratulus lacteus; Crfor, Crepidula fornicata; Cv, Chaetopterus variopedatus; Dm, Drosophila melanogaster; Dr, Danio rerio; Em, Echinococcus multilocularis; Hs, Homo sapiens; Hv, Hydra vulgaris; Kram, Kronborgia amphipodicola; Mm, Mus musculus; Osc, Oscarella carmela; Pd, Platynereis dumerilii; Ph, Parhyale hawaiensis; Pnig, Polycelis nigra; Pten, Polycelis tenuis; Sci, Sycon ciliatum; Sk, Saccoglossus kowalevskii; Sm, Schistosoma mansoni; Smed, Schmidtea medi [file pgen.1007030.s010.tif]
