# Supplementary material for: A C-terminally truncated form of β-catenin acts as a novel regulator of Wnt/β-catenin signaling in planarians
Source: PLoS Genet. 2017 Oct 4;13(10):e1007030. doi: 10.1371/journal.pgen.1007030 (PMC5643146; doi:10.1371/journal.pgen.1007030)
Supplement: S11 Fig — The functional domains are indicated. Arm splicing isoform (Neural Arm, accession number AAB58731.1) and Plakoglobin, highlighted in yellow, conserve all functional domains but show a shorter C-terminal transactivation domain. Accession numbers and abbreviations are indicated in S10 Fig. (PDF) [file pgen.1007030.s011.pdf]

Align bcatenins- Supp Inf

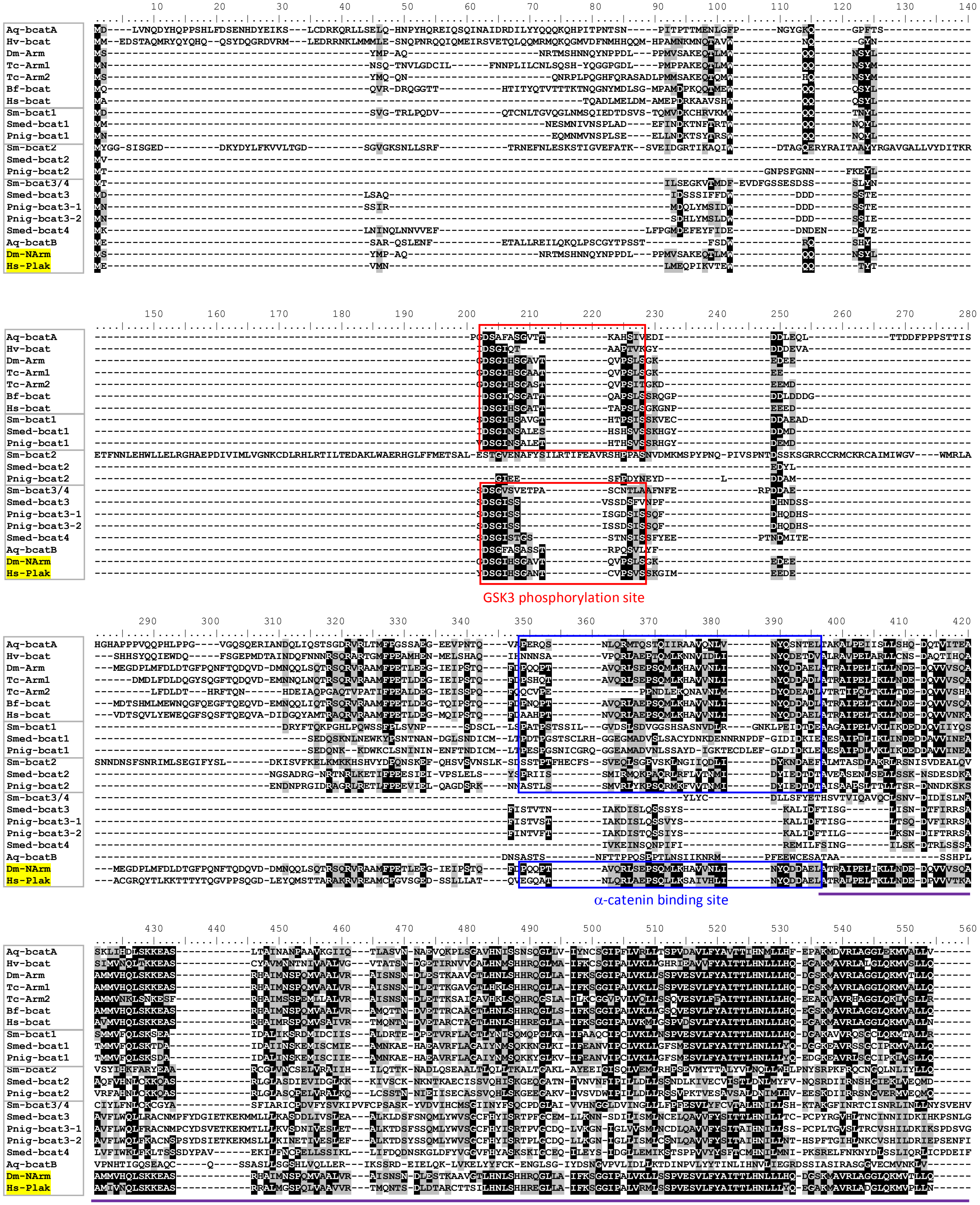

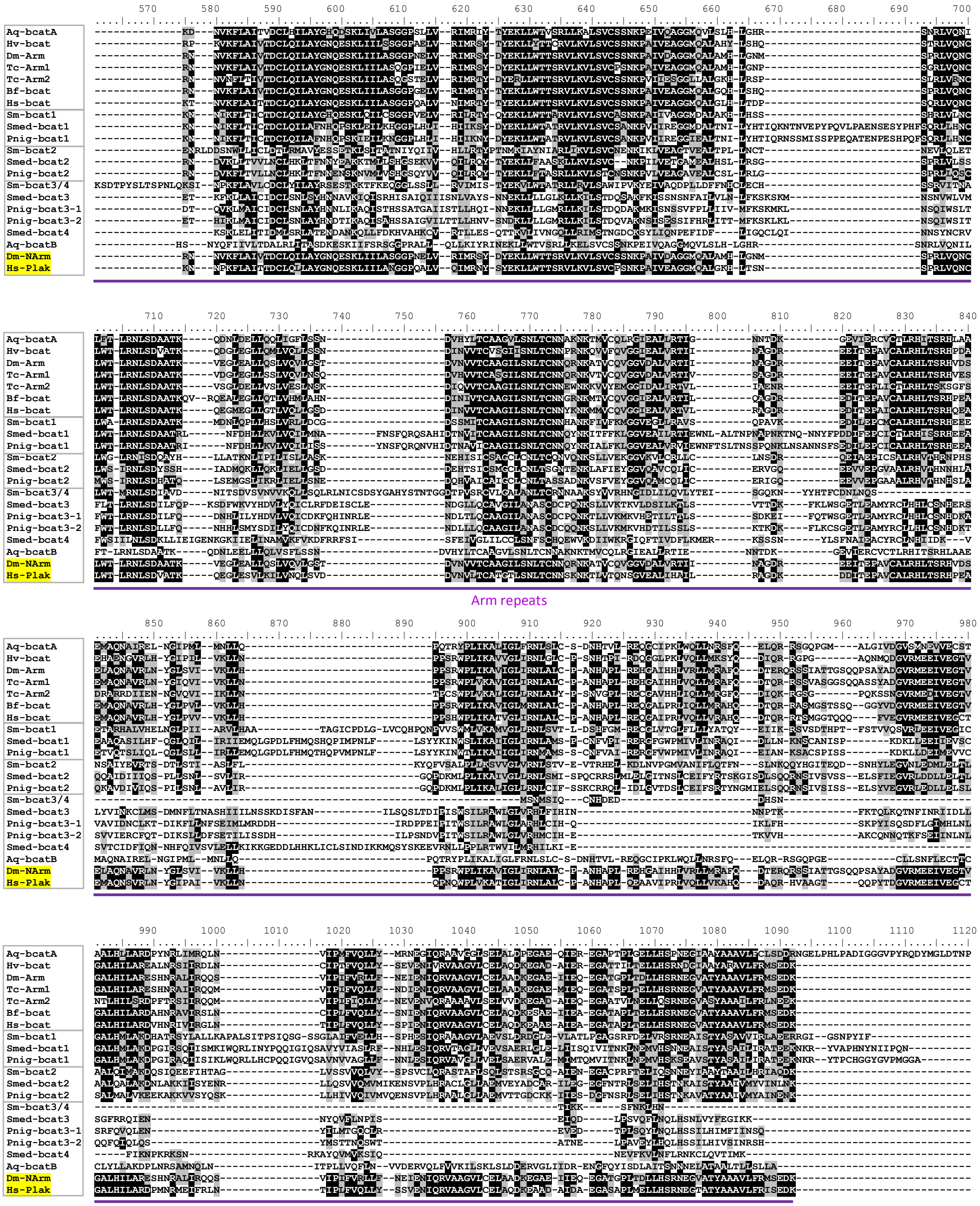
Aq-bcatA
Hv-bcat
Dm-Arm
Tc-Arm1
Tc-Arm2
Bf-bcat
Hs-bcat
Sm-bcat1
Smed-bcat1
Pnig-bcat1
Sm-bcat2
Smed-bcat2
Pnig-bcat2
Sm-bcat3/4
Smed-bcat3
Pnig-bcat3-1
Pnig-bcat3-2
Smed-bcat4
Aq-bcatB
Dm-NArm
Hs-Plak
Arm repeats

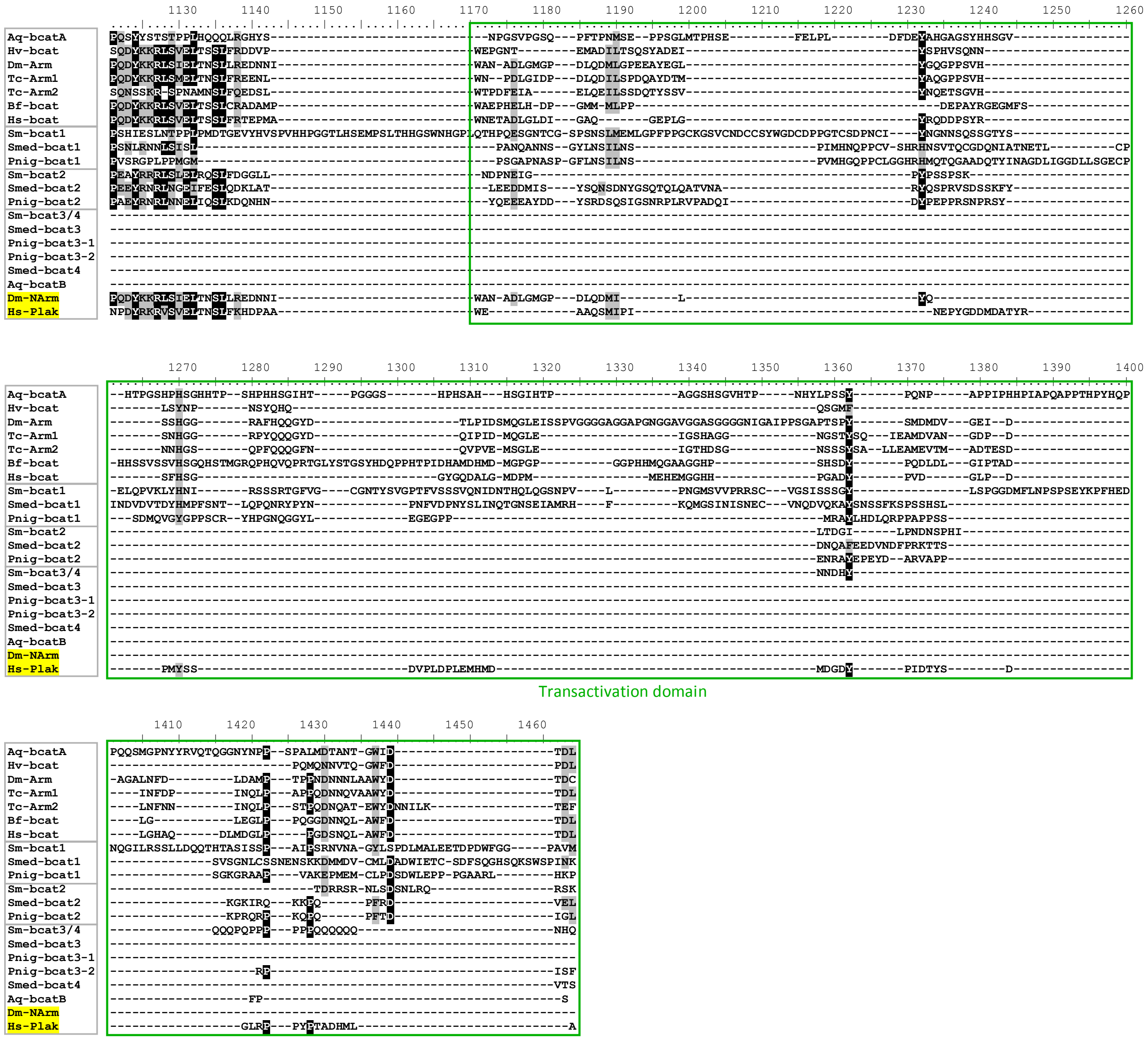
1130 1140 1150 1160 1170 1180 1190 1200 1210 1220 1230 1240 1250 1260
1270 1280 1290 1300 1310 1320 1330 1340 1350 1360 1370 1380 1390 1400
1410 1420 1430 1440 1450 1460
Aq-bcatA
Hv-bcat
Dm-Arm
Tc-Arm1
Tc-Arm2
Bf-bcat
Hs-bcat
Sm-bcat1
Smed-bcat1
Pnig-bcat1
Sm-bcat2
Smed-bcat2
Pnig-bcat2
Sm-bcat3/4
Smed-bcat3
Pnig-bcat3-1
Pnig-bcat3-2
Smed-bcat4
Aq-bcatB
Dm-NArm
Hs-Plak
Transactivation domain
