# Supplementary material for: A C-terminally truncated form of β-catenin acts as a novel regulator of Wnt/β-catenin signaling in planarians
Source: PLoS Genet. 2017 Oct 4;13(10):e1007030. doi: 10.1371/journal.pgen.1007030 (PMC5643146; doi:10.1371/journal.pgen.1007030)
Supplement: S1 Table — (PDF) [file pgen.1007030.s016.pdf]

gene	Primer sequences	
	**for probe**	**for dsRNA**
ƀ-catenin1	Iglesias et al. 2008	Iglesias et al. 2008
ƀ-catenin3	5ôATGGATTTAAGTGCTCAAATCGATTCGTCATC-3ô	5ôTGCTCTATTGGGTGAGCGGATGTTTTCA-3ô
	5ôCATTAATCAAGAGGTAGAAAATGAAGTTTGTTTG-3ô	5ôACTCCCCGATACGGACAGGGACAGGT-3ô
ƀ-catenin4	5ôTTTGCTGGCTTTGATGAAACTAAATATAAATC-3ô	5ôAAGCATGTCGCTCACAAATGCGTTCG-3ô
	5ôTCATGAAGTTACCTTCATAATGGTTACCTG-3ô	5ôTTCTCCGATTTCAATCAGCAGCTTATCGG-3ô
TCF-1	5ôTCACCATTGAAGCCCACTCC-3ô	
	5ôTAGGTGGTACCGAAGGCTCA-3ô	
TCF-2	5ôATGGCATGAGTTGTCTCGGG-3ô	5ôTGCGCCATGCTCGAATAGAT-3ô
	5ôACTGGCGGTTTGATCGTCAT-3ô	5ôACTGGCGGTTTGATCGTCAT-3ô
TCF-3	5ôGCGTTGACTCGAGAAGACCA-3ô	
	5ôGGTCAGCCGATAGATTCCCG-3ô	
APC-1		Iglesias et al. 2010

gene	Primer sequences
	**For Real time PCR**
ƀ-catenin1	5'-CAAGAGCCCATCATCTCA-3'
	5'-CGTCCAACATACATACGTCC-3'
ƀ-catenin2	5'-GGCACTTCGTCATGTTACC-3'
	5'-CACGAATCAGCACCGATA-3'
ƀ-catenin3	5'-TCGACCACAATGACAGTTCT-3'
	5'-TGTGAAATCGATGAGTGCTT-3'
ƀ-catenin4	5'-TACCTCCTCTTCGCACTTG-3'
	5'-ACTTGGTAGGCTTTTCGATTT-3'
APC-1	5'-CAAACGGATGGCGTTAC-3'
	5'-ATGAGGAACTACTGCGGG-3'
TCF-2	5'-CATGGCAAAATCAAACAGG-3'
	5'-CGATAAACGAAGGGGTTCT-3'
URA4	5'-TTCACGTTGTCGATCTAGCC-3'
	5'-CGAATATCCTCTGCCAGTGC-3'
